# Immunogenicity of a trivalent haemorrhagic fever vaccine candidate against Sudan virus, Marburg virus and Lassa virus in an mpox vaccine

**DOI:** 10.1099/jgv.0.002157

**Published:** 2025-10-06

**Authors:** Martina Pfranger, Nina Krause, Benedikt Asbach, Johannes Meier, George Carnell, Lara Scheer, Anja Kalender, David Brenner, Paul Tonks, Simon Frost, Edward Wright, Ingo Jordan, Emma Kennedy, Roger Hewson, Barbara Blacklaws, Andrew Chan, Srivatsan Parthasarathy, Stuart Dowall, Miles Carroll, Volker Sandig, Sofiya Fedosyuk, Rebecca Kinsley, Jonathan Heeney, Ralf Wagner

**Affiliations:** 1 Institute of Medical Microbiology and Hygiene, University of Regensburg, Regensburg, Germany; 2 Department of Veterinary Medicine, Lab of Viral Zoonotics, University of Cambridge, Cambridge, UK; 3 One Virology, Wolfson Centre for Global Virus Research, School of Veterinary Medicine and Science, University of Nottingham, Nottingham, UK; 4 DIOSynVax Ltd™, Cambridge, UK; 5 Microsoft Health Futures, Redmond, USA; 6 London School of Hygiene and Tropical Medicine, London, UK; 7 School of Life Sciences, University of Sussex, Brighton, UK; 8 ProBioGen AG, Berlin, Germany; 9 UK Health Security Agency (UK), Porton Down, Salisbury, UK; 10 Pandemic Sciences Institute & Centre for Human Genetics, University of Oxford, Oxford, UK; 11 Institute of Clinical Microbiology and Hygiene, University Hospital Regensburg, Regensburg, Germany

**Keywords:** haemorrhagic fever viruses, Modified Vaccinia Ankara (MVA), multivalent vaccine

## Abstract

A multivalent vaccine targeting high-consequence infectious diseases in Sub-Saharan Africa (SSA), which are linked to high mortality, morbidity and overlapping clinical manifestations, would significantly improve health security and economic stability in this region. Trivalent vector vaccines were devised to deliver digitally optimized versions of Orthoebolavirus, Orthomarburgvirus glycoproteins (GPs) and a Lassa mammarenavirus (LASV) nucleoprotein (NP) by a single Modified Vaccinia Ankara (MVA) known to protect against mpox virus (MPXV) along with a matched DNA vaccine. Three immunizations in mice and Hartley guinea pigs with MVA only or a DNA prime followed by two MVA administrations induced comparable levels of binding antibodies and LASV-specific T-cells, respectively. While DNA priming mitigated MVA-specific antibody responses, GP- and NP-specific antibodies developed already after a single MVA vaccination. Although a post-outbreak Ebola virus vaccine is available, outbreaks by other filoviruses, annual LASV epidemics and increased incidence of MPXV infections support the rationale for an MVA-based trivalent haemorrhagic fever vaccine for endemic and high-risk human populations in SSA.

**Impact Statement**

Orthoebolaviruses, Orthomarburgviruses and Mammarenaviruses continue to cause severe haemorrhagic fevers with overlapping symptoms and high case fatality rates in West and Central Africa. At the same time, the increasing incidence of mpox further highlights the need for a multivalent haemorrhagic fever vaccine. This study introduces a trivalent vaccine based on the Modified Vaccinia Ankara platform, designed to protect against all three haemorrhagic fever viruses while also offering potential protection against mpox. By delivering broad immune protection through a single, cost-effective, scalable vaccine, this approach offers a powerful tool for improving outbreak preparedness, reducing mortality and strengthening health security in Sub-Saharan Africa.

## Introduction

Viral haemorrhagic fevers (VHFs) caused by re-emerging zoonotic RNA viruses such as Orthoebolaviruses, Orthomarburgviruses and Lassa viruses pose a serious public health threat in West and Central Africa due to their high case fatality rates and epidemic potential [1]. Following zoonotic spillover, VHFs spread rapidly via human-to-human transmission and may reach non-endemic areas through travel. Climate and environmental changes and shifting migratory dynamics of reservoir species are thought to increase spillover risk and expand endemic regions, making outbreaks more difficult to control [2,3].

Orthoebolaviruses and Orthomarburgviruses belong to the family of filoviruses and are primarily transmitted by migrating bats and were responsible for the most severe outbreaks in humans and non-human primates (NHPs). The 2013–2016 West African epidemic caused by Ebola virus (EBOV; *Orthoebolavirus zairense*) resulted in over 28,000 cases and 11,000 deaths [4,5]. In 2022, an outbreak of Sudan virus (SUDV; *Orthoebolavirus sudanense*) led to 142 confirmed cases and 55 deaths with an overall case fatality rate of 39% [6,7]. Outbreaks with Marburg virus (MARV; *Orthomarburgvirus marburgense*) are less frequent but severe across Sub-Saharan Africa (SSA), with case fatality rates ranging from 24% to 88%. Recently, new cases were reported for the first time in Equatorial Guinea, Rwanda or Tanzania [8,9].

Lassa virus (LASV), an Old World arenavirus (*Mammarenavirus lassaense*) endemic in West Africa, is transmitted via contact with infected rodent urine or faeces. LASV causes ~500,000 Lassa fever (LF) infections and over 5,000 deaths annually with a mortality rate of around 15–30% among hospitalized patients [10].

Filoviruses and arenaviruses co-circulate in overlapping regions of West Africa, which poses significant challenges for clear diagnosis. During the 2013–2016 Ebola outbreak, early cases were misdiagnosed as LF, delaying the response and accelerating the spread of EBOV [11]. All three VHFs are WHO blueprint priority pathogens, highlighting the need for a broadly protective, multivalent haemorrhagic fever vaccine.

Currently, two EBOV vaccines are approved: Ervebo^®^, a single-dose, replication-competent vesicular stomatitis virus (VSV)-based vaccine expressing the EBOV glycoprotein (GP). Zabdeno/Mvabea^®^ is a heterologous prime-boost regimen combining an adenovirus serotype 26 vector expressing the EBOV GP Mayinga/1976 with MVA-BN-Filo, which encodes GPs derived from EBOV, SUDV and MARV, as well as the nucleoprotein (NP) of Taï Forest virus (TAFV) [12,13]. Therapeutics like Inmazeb^®^ and Ebanga^®^ are also approved for treating infections caused by EBOV [14].

Ervebo^®^ is highly efficacious but only approved for EBOV and is associated with adverse events such as high temperature and vaccine-induced arthritis [15,17]. Zabdeno/Mvabea^®^ is multivalent by design but is only approved against EBOV so far [18,19].

Due to the risk of recurrent outbreaks and viral spread beyond SSA, we aimed to develop a multivalent vaccine inducing cross-protective immunity against Old World arenaviruses and filoviruses.

We selected Modified Vaccinia Ankara (MVA) as our multivalent delivery platform due to its excellent safety profile and strong immunogenicity, even in immunocompromised individuals and children [20,21]. MVA is widely used in vaccines targeting infectious diseases [22], cancer [23] and smallpox [24]. MVA also provides protection against MPXV [25,26] and is WHO-prequalified (JYNNEOS/IMVANEX) for mpox [27]. Notably, the recent increase in mpox cases in West Africa further supports the use of MVA in this region.

In this study, we constructed two trivalent vaccine candidates, MVA-HFVac3.v1 and DNA-HFVac3.v1, delivering digitally optimized GPs derived from Orthoebolaviruses and Orthomarburgviruses along with NP from Lassa virus isolates. HFVac3.v1 alone induces potent, pan-filovirus and LASV NP-specific immune response and MPXV cross-reactive antibodies in mice and Hartley guinea pigs. Given the re-occurring outbreaks by various filoviruses, annual LASV epidemics and an increased incidence of MPXV infections support the rationale for an MVA-based trivalent haemorrhagic fever vaccine for endemic and high-risk human populations.

## Methods

### Cells

BHK-21 (ATCC CCL-10), DF-1 (ATCC CRL-12203) and HEK293T cells were cultivated in Dulbecco’s MEM (DMEM) supplemented with 10% FCS and 1% penicillin/streptomycin (Pen/Strep) at 37 °C and 5% CO_2_ in a humidified incubator.

Adherent AGE1.CR.pIX cells (ProBioGen AG, Berlin) [28] were grown in DMEM-F12 medium (Cat # 10565-018, Thermo Fisher Scientific, Germany) supplemented with 5% bovine serum (Cat # 12003C, γ-irradiated, Sigma Aldrich/Merck, Germany).

### Immunogen design and codon optimization

The monovalent DNA and MVA reference vaccines encode full-length GP sequences from EBOV/Yambuku-Mayinga/1976 [E-GP.wt, aa 1–676, excluding the mucin-like domain (ΔMLD), aa 314–464, GenBank: NC_002549.1], MARV/Mt.Elgon-Musoke/1980 (M-GP.wt, aa 1–681, GenBank: NC_001608) and the full-length NP sequence of LASV/GA391.Nigeria/1977 (L-NP.wt, aa 1–490, GenBank: X52400.1), respectively.

To generate optimized antigens, representative DNA sequences from the most clinically relevant and outbreak-associated strains of Orthoebolavirus, Orthomarburgvirus and Lassa virus isolates were retrieved from public databases. These obtained sequences were aligned in codon space, and maximum likelihood phylogenies were reconstructed to guide the initial development of optimized consensus sequences. Following *in silico* translation, multiple sequence alignment was generated using the MAFFT algorithm [29] with global alignment (‘ginsi’) and default parameters. Maximum phylogenetic trees were reconstructed using IQTREE v2.2.0 [30] under default settings, which performs model selection prior to reconstructing the phylogeny [31]. The resulting engineered variants were further refined *in silico* based on selected immunodominant epitopes predicted to elicit robust B- and T-cell responses, including but not limited to adjustments to glycosylation patterns, insertions/deletions and point mutations, while maintaining structural integrity using a proprietary pipeline (DIOSynVax Ltd). For example, in the case of the Orthoebolavirus antigen, the mucin-like domain was depleted, as prior studies have shown that this region contributes to cytotoxicity and can mask key epitopes from immune recognition. All DNA sequences were subsequently codon-optimized for efficient expression in human cells using the GeneArt^™^ optimization tool. Codon optimization was performed to enhance translational efficiency by modifying the nucleotide sequences to preferentially use codons that are more efficiently translated by the human host cell machinery, without altering the resulting amino acid sequence [32].

Final constructs encode optimized GP sequences of Orthoebolaviruses (E-GP.v1, aa 1–671, ΔMLD aa 314–464, GenBank: KT878488) and Orthomarburgviruses (M-GP.v1, aa 1–490, GenBank: NC_001608) along with an NP sequence of different Lassa virus isolates (L-NP.v1, aa 1–490, GenBank: nr X52400.1), respectively.

### Construction and preparation of DNA vaccines

Wildtype (E-GP.wt, M-GP.wt and L-NP.wt) and digitally designed (E-GP.v1, M-GP.v1 and L-NP.v1) antigen sequences were codon-optimized for expression in human cells using the GeneArt^®^ GeneOptimizer^®^ (Thermo Fisher Scientific, Germany). Sequences were synthesized by GeneArt (Thermo Fisher Scientific) and cloned into the DNA expression vector pURVac, which includes a strong human CMV promoter in combination with a human T-cell leukaemia virus-1 regulatory and splicing element and a bovine growth hormone poly-A terminator. For the trivalent DNA vaccine vector HFVac3.v1, the ORFs encoding L-NP.v1, M-GP.v1 and E-GP.v1 were linked in that order via thosea asigna virus 2A (T2A) and porcine teschovirus-1 2A (P2A) sites by fusion PCR and cloned into the pURVac expression vector.

In order to interfere to the least possible extent with intracellular protein trafficking, we decided to start translation with the cytoplasmic NP, followed by the two filovirus GPs known to be transported via ER and Golgi compartments to the cell surface. As previous studies have shown that full-length Orthoebolavirus GPs are often cytopathic, inducing cell death through endoplasmic reticulum stress and activation of the unfolded protein response [33,35], efforts were undertaken to reduce cytotoxicity by using a digitally designed version of the mucin-like domain-depleted GP and placing it at the 3′ part of our polycistronic construct. All cloned constructs were sequence-verified and purified using the EndoFree Plasmid Mega Kit (Qiagen, Hilden, Germany).

### Construction, purification and growth of recombinant MVAs

Antigen sequences for MVA vaccine vectors were codon-optimized for enhanced expression in human cells using the GeneArt^®^ GeneOptimizer^®^ and further modified by removal of termination signals of vaccinia virus-specific early transcription and extended oligo C, G and GC, as well as oligo A, T and AT stretches [36,37].

For monovalent reference MVAs (MVA E-GP.wt, MVA M-GP.wt and MVA L-NP.wt), antigen sequences were cloned into the MVA shuttle vectors with recombination flanks for the viral TK integration locus, respectively, under the transcriptional control of a short synthetic early/late (SSP) promoter. Recombinant MVAs were generated via homologous recombination and plaque-purified, and virus stocks were produced as described previously [38].

The trivalent MVA-HFVac3.v1 was generated in a stepwise process using two distinct MVA integration sites for homologous recombination. L-NP.v1 under the mH5 promoter was inserted into the deletion site III (Del III) of MVA, followed by an integration of a dual-promoter expression of E-GP.v1 and M-GP.v1 (driven by p7.5 and SSP promoters) into the TK locus of MVA. Recombinant viruses were plaque-purified, propagated on AGE1.CR.pIX cells and purified by sucrose gradient ultracentrifugation as described in [39,40]. Final titres were determined via crystal violet staining on DF-1 [41].

### Transfection of DNA vaccine vectors and infection of rMVAs

For *in vitro* characterization, 5×10^5^ HEK293T cells were transfected with 2.5 µg DNA and 7.5 µg polyethylenimine per 6-well plate [42]. After 6 h in serum-free DMEM, the medium was replaced with complete DMEM (10% FCS, 1% Pen/Strep). Cells were harvested 48 h post-transfection for Western blot or surface staining.

For MVA infection, HEK293T cells were infected at MOI 2 in serum-free DMEM with a change to complete medium after 2 h. Cells were collected 24 h post-infection for expression analysis.

### Expression of MARV GP-specific human monoclonal antibody MR78 for Western blot analysis

For Western blot analysis, the human monoclonal antibody MR78 was produced in-house as described in [43]. For cloning, the monoclonal antibody light-chain and heavy-chain variable domain sequences were retrieved from RSCB PDB (5JRP) [44]. The sequences were codon-optimized and synthesized by GeneArt AG (Thermo Fisher Scientific, Germany) and cloned into human IgG heavy-chain or kappa light-chain expression vectors, respectively.

The human monoclonal antibody MR78 was transiently expressed in Expi293F cells (Thermo Fisher Scientific, USA) according to the manufacturer’s instructions. The antibodies were purified from the supernatants via protein A-based affinity purification. Elution from the column was accomplished by a pH step using 100 mM glycine buffer pH 3.2, and the eluted antibody was immediately buffer-exchanged to dPBS.

### Western blot analysis

Western blot analysis was performed as previously described [40]. Cells were lysed with TDLB buffer (50 mM Tris, pH 8.0, 150 mM NaCl, 0.1% SDS, 1% Nonidet P-40, 0.5% sodium deoxycholate) with protease inhibitors (Complete Mini, Roche, Basel, Switzerland), and protein concentrations were determined via Bradford assay (Protein Assay, BioRad, Germany). Proteins were separated on 12.5% SDS-PAGE under reducing conditions and transferred to nitrocellulose membranes. Membranes were stained with primary antibodies anti-MARV GP (in-house produced, human monoclonal antibody MR78, 1 : 100), anti-EBOV GP (Cat. # 40304-T46, 1 : 1000, Sino Biological, China), anti-LASV NP (Cat. # 01-04-0104, 1 : 1000, Cambridge Biologics, USA), anti-2A peptide (Cat. # MABS2005, 1 : 1000, Sigma Aldrich/Merck, Germany), anti-vaccinia protein (Cat. # 1-VA003-07, 1 : 5000, Quartett) and anti-GAPDH (Rabbit, CST, 1 : 2500) followed by staining using either goat anti-mouse (Cat. # 115-036-003, 1 : 10000, Jackson, USA), goat anti-rabbit (Cat. # P0448, 1 : 2000, Dako, USA) or rabbit anti-human (Cat. # P0214, 1 : 2000, Dako, USA) IgG/HRP-conjugated secondary antibodies. Femto ECL substrate (Thermo Fisher Scientific, USA) was used for protein detection with the Chemilux Pro imaging system (Intas, Germany).

### Surface staining and flow cytometry analysis

For flow cytometry, transfected or rMVA-infected cells were harvested after 48 or 24 h, respectively. Surface staining was performed using 10 µg /ml of Orthoebolavirus GP-trimer specific (CA45) or Orthomarburgvirus GP-specific (MR186) antibodies in FACS buffer (PBS, 1% inactivated FCS, 2 mM EDTA) for 1 h. After washing, cells were stained with Alexa-647–conjugated goat anti-human IgG secondary antibody 20 µg/ml (Catalogue # A-21445, Thermo Fisher Scientific, USA) in FACS buffer for 1 h in the dark, followed by additional washing. Samples were analysed on an Attune NxT cytometer. Gating included controls for untransfected/uninfected and empty vector-transfected or wt MVA-infected cells. Data analysis was conducted using Attune NxT software and GraphPad Prism. Cells were washed again with FACS buffer three times, followed by flow cytometry analysis using the Attune NxT device. Cells were gated on stained and untransfected/uninfected, as well as wt MVA-infected or empty DNA-transfected cells. The evaluation of the data was performed using Attune NxT software and GraphPad Prism.

## Animal experiments

Animal studies were approved by the Animal Welfare and Ethical Review Body, University of Cambridge, and experiments were carried out under an approved UK Home Office Licence (P814342B and PP9157246). All animals were monitored daily. For terminal bleeds, animals were anesthetized using isoflurane.

**Fig. 1.**
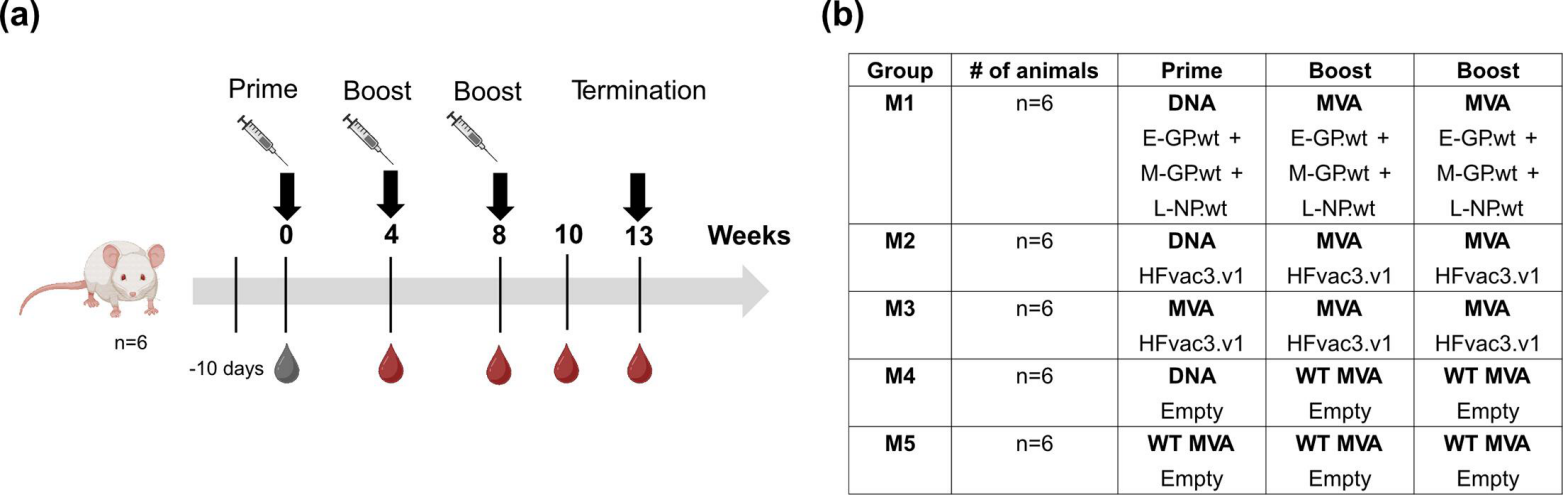
Immunization schedule and vaccination groups. (**a**) Serum samples were collected from all animals prior to any immunization and at the indicated time points (weeks) depicted as blood drop symbols (timeline and experimental design created with BioRender.com). The study was terminated 5 weeks after the third immunization. (**b**) BALB/c mice were primed at week 0 either with a mixture of DNA vaccine vectors each encoding one of the three indicated wt antigens (group 1), or with a trivalent DNA vaccine vector (DNA HFVac3.v1; group M2) via the subcutaneous route (s.c.), or with the trivalent MVA (MVA HFVac3.v1, group M3) via the intramuscular (i.m.) route. Primed mice were boosted i.m. at weeks 4 and 8 either with the mixture of rMVAs encoding each one of the indicated antigens (group M1) or the trivalent MVA-HFVac3.v1 (groups M2 and M3), respectively. As a control, animals were primed with an empty DNA (group M4) or WT MVA (group M5) and boosted with WT MVA, respectively.

Mouse study: Five groups of six female BALB/c mice (8–10 weeks old, Charles River) were immunized with DNA vaccines subcutaneously (50 µg per construct, s.c., rear flank) or MVA vaccines via the i.m. route (1×10⁷ p.f.u., i.m., hind legs) using a total volume of 50 µl per injection site. Blood was collected from the saphenous vein at designated time points (Fig. 1). Five weeks after the final immunization, mice were terminally bled via cardiac puncture.

Guinea pig study*:* Four groups of four female Hartley guinea pigs (7–8 weeks old, Envigo) were immunized at week 0 with 200 µg DNA vaccine via the i.d. using the PharmaJet^©^ device (i.d., PharmaJet^®^, hind legs) in a total volume of 200 µl. MVA vaccines (2×10⁷ p.f.u., i.m.) were administered at weeks 4 and 12 via the i.m. route in a total volume of 100 µl. Bleeds were taken via the saphenous vein, and terminal samples were collected by cardiac puncture under non-recovery anaesthesia. Euthanasia was performed with pentobarbital under non-recovery anaesthesia.

### Luminex binding antibody assay

Antigens were covalently coupled to Luminex MagPlex® beads using EDC/S-NHS chemistry in MES buffer (pH 6.0), following a modified protocol from the xMAP^®^ Cookbook (Fifth Edition, Luminex). Coupling was performed with 12.5 µg of recombinant protein per 1.25×10⁷ beads. The following recombinant proteins were used: SUDV GP (IBT Bioservices, #0502-001), EBOV GP (IBT Bioservices, #0501-001), MARV GP (IBT Bioservices, #0503-001), RAVV ΔMuc GP (IBT Bioservices, #0513-015), LASV NP (LASV NP; Zalgen, #LASV-R-0031), MPXV A35 (Stratech, #40886-V08H-SIB,), MPXV B6 (Stratech, #40902-V08H-SIB), MPXV H3 (Sino Biologicals, #40893-V08H1) and MPXV M1 (Stratech, #40904-V07H-SIB). Coupled beads were stored in PBS with 1% BSA and 0.05% sodium azide at 4 °C.

Serum samples were diluted (1 : 100, 2-fold, 8-point series) in PBS with 1% milk powder and incubated with coupled bead mixes in black 96-well plates for 1 h at 37 °C, 600 r.p.m. After washing in PBS-T (PBS+0.05% Tween-20), beads were incubated with PE-conjugated goat anti-mouse IgG (H+L) cross-adsorbed (Invitrogen, #P852) or donkey anti-guinea pig F(ab’)2 fragment (Jackson ImmunoResearch, #706-116-148) in Luminex assay buffer (PBS with 1% BSA, 0.05% NaN₃, 0.5% PVA and 0.8% PVP) for 30 min at 37 °C. Plates were washed, resuspended in PBS-T and read on a Luminex-200 analyzer with the default setting of measuring the fluorescence intensity of 50 beads per colour/target. Results are given as mean fluorescence intensity (MFI).

To ensure consistency across assay runs, MFI values on each plate were normalized to an internal positive reference control serum, which was included alongside the test samples on every plate. Additionally, each plate contained two blank control wells (no serum added) to monitor background signal and assess non-specific binding.

Specificity of the Luminex assay was confirmed by testing 36 negative serum samples from mice and 40 negative serum samples from guinea pigs. Sensitivity was determined using 36 positive serum samples from mice and 40 positive serum samples from guinea pigs by calculating the limit of detection for EBOV GP, SUDV GP, MARV GP, RAVV GP and LASV NP. For the MPXV antigens, the sensitivity, specificity and reproducibility analyses were restricted to guinea pig samples due to the low amount of murine serum samples. Results were analysed using receiver operating characteristic (ROC) curve analysis to evaluate the diagnostic performance of the assay. The ROC curves were generated to determine the sensitivity and specificity across a range of thresholds. Confidence intervals for sensitivity, specificity and AUC were calculated using the Wilson/Brown method. All analyses were performed using GraphPad Prism 10. Reproducibility was assessed by analysing positive control samples in replicates within and across assay runs. Variability between plates was consistently less than 10% demonstrating high reproducibility. Specificity, sensitivity and accuracy were calculated for mouse and guinea pig antisera for the various antigens used in the Luminex assay (Tables S1 and S2).

NIBSC standards were not included, as no commercially available standards exist for mice or guinea pigs.

### Detection of MVA-specific antibodies by ELISA

BHK-21 cells were infected with WT MVA at MOI 0.1 for 24 h, washed with PBS and lysed in PBS containing 1% Triton X-100 and protease inhibitor (Roche, #11697498001).

Cell lysate was diluted 1 : 10 in 0.05 M carbonate/bicarbonate buffer (pH 9.6), and 96-well Maxisorp plates were coated overnight at 4 °C (100 µl per well). Plates were washed with PBS+0.05% Tween-20 and blocked with PBS+3% milk powder for 1 h at room temperature. Serum samples were diluted (8-point, 2-fold, starting at 1 : 100) in blocking buffer and incubated for 1 h at room temperature, shaking at 450 r.p.m. After washing, HRP-conjugated anti-mouse IgG (Abcam, #ab6829; 1 : 10,000) was added for 1 h. Plates were developed with 1-Step Ultra TMB (10 min, dark), stopped with 2N H₂SO₄ and read at 450 nm with a 630 nm reference using a BioTek plate reader.

### Production of pseudotyped lentiviral vectors

Lentiviral pseudotypes were produced by transient transfections of HEK293T/17 cells (4×10^5^ cells/6-well) with lentiviral packaging plasmids p8.91-gag-pol and pCSFLW-firefly luciferase [45,46] and the DNA plasmid encoding the respective GP (SUDV GP/Gulu/2000, GenBank: NC_006432; SUDV GP/Uganda/2022, GenBank: OQ672950.1; EBOV GP/Yambuku/1976, GenBank: NC_002549; MARVGP/Musoke/1980, GenBank: NC_001608) using the FuGENE^®^-HD Transfection Reagent (Promega). Supernatant was harvested after 48 h, filtered through a 0.45 µm filter, aliquoted and immediately stored at −80 °C.

A single, well-characterized batch of pseudotyped virus was generated for each filovirus GP and used across all neutralization assays presented in this manuscript to ensure consistency of our data. Each aliquot was defrosted only once and discarded after use. Pseudotyped viruses were titrated on HEK293T cells.

### Pseudotype microneutralization assay

Pseudotype-based microneutralization assays were performed as described previously [47]. Briefly, serial dilutions (1 : 2) of serum (1 : 33) were incubated with SUDV GP/Gulu/2000, SUDV GP/Uganda/2022 and EBOV GP/Mayinga/1976 or MARV GP/Musoke/1980-bearing lentiviral pseudotypes, respectively, for 1 h at 37 °C and 5% CO_2_ in white 96-well culture plates. HEK293T/17 cells (1.5×10^4^) were then added per well, and plates were incubated for 48 h at 37 °C and 5% CO_2_ in a humidified incubator. Bright-Glo (Promega) was then added to each well, and luminescence was read after 5 min incubation using the GloMax microplate reader (Promega). Experimental data points were normalized to 100 and 0% neutralization. Normalized responses were plotted against the log10(dilution factor) and fitted to the nonlinear model ‘log(inhibitor) vs normalised response – Variable slope’.

### ELISpot

Murine splenocytes were harvested, cryopreserved in FBS containing 10% DMSO and stored in liquid nitrogen.

LASV NP-specific T-cell responses were assessed using the Mouse IFN-γ ELISpotPLUS kit (CTL ImmunoSpot, USA) according to the manufacturer’s instructions. Briefly, thawed splenocytes were plated in pre-coated 96-well ELISpot plates at a density of 2–5×10⁵ cells/well and stimulated with overlapping 15-mer peptide pools (11 amino acid overlap) corresponding to the vaccine antigen sequences. After 18–24 h incubation at 37 °C, plates were washed and developed using biotinylated detection antibody, streptavidin-HRP and substrate. Plates were air-dried for 24 h. Spots were quantified using an ImmunoSpot S6 Ultra M Analyzer (CTL) and analysed with ImmunoSpot Software v7.0.22.1.

### Statistical analysis

Data were analysed using GraphPad Prism software version 9.2.0. Non-parametric, two-tailed Mann–Whitney U tests were performed for all the pairwise comparisons.

## Results

### Design and biochemical characterization of a trivalent single-vector HFVac3.v1 MVA and DNA vaccine

To guide antigen design, GP sequences from the most clinically relevant and outbreak-associated Orthoebolaviruses and Orthomarburgviruses, as well as NP sequences from Lassa virus isolates, were subjected to *in silico* optimization.

Phylogenetic trees illustrating the distances of the Orthoebolavirus-derived GP (E-GP.v1), the Orthomarburgvirus-derived GP (M-GP.v1) and the Lassa mammarenavirus-derived NP (L-NP.v1) to their respective wt reference sequences are shown in Fig. S1, available in the online Supplementary Material, and protein homology is detailed in Fig. S2. In line with the phylogenetic relationship, E-GP.v1 revealed the highest protein homology to the SUDV GPs, M-GP.v1 shows the best homology to MARV GP Musoke and RAVV (Ravn virus) GP and L-NP.v1 shows generally good homology to the highly conserved NPs of other Lassa viruses, particularly LASV NP lineage II.

**Fig. 2.**
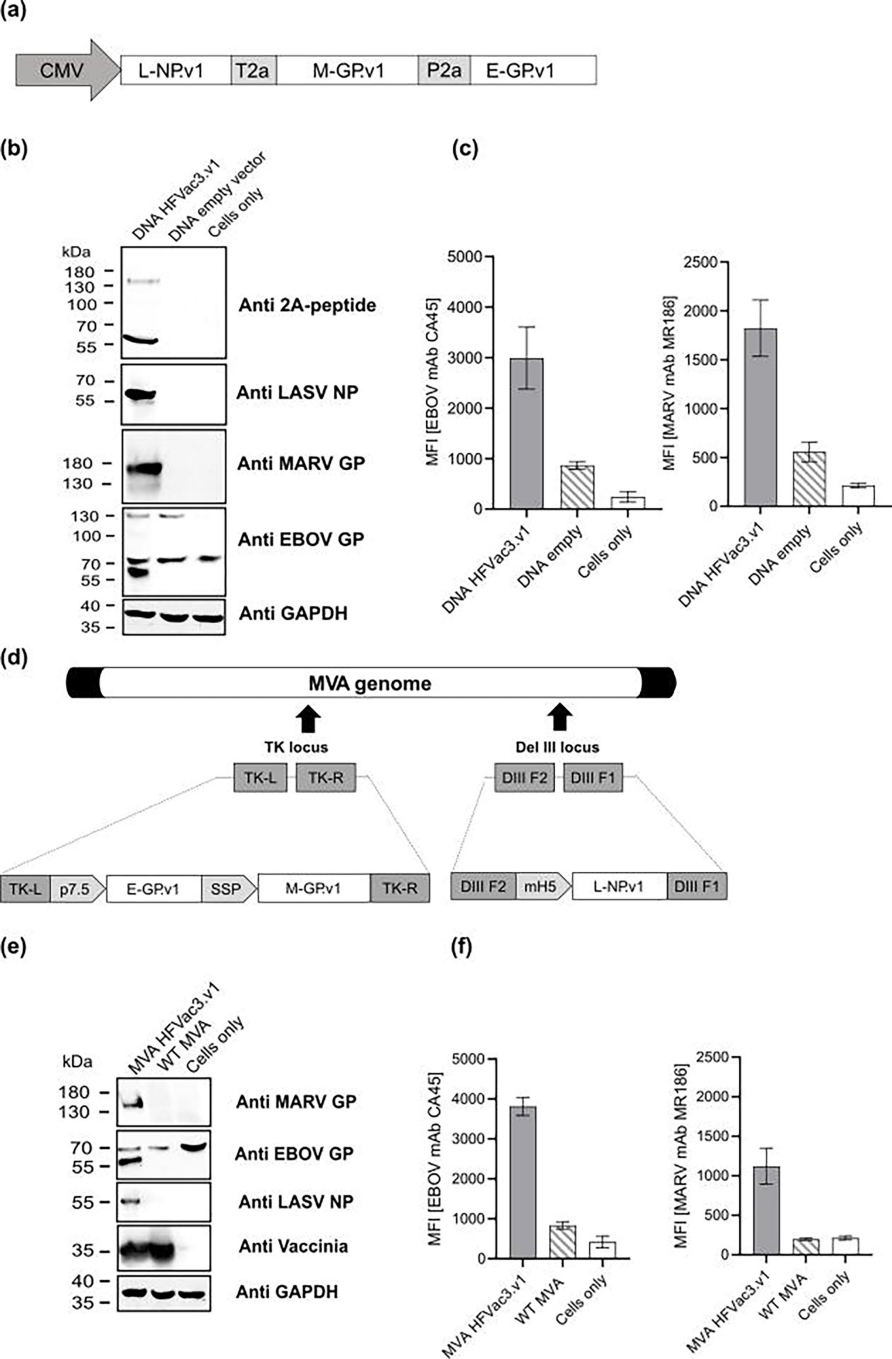
Antigen design and characterization of multivalent DNA- and MVA-based vaccine candidates HFVac3.v1. (**a**) Schematic of DNA-HFVac3.v1 encoding L-NP.v1, M-GP.v1 and E-GP.v1 linked via T2A/P2A under a CMV promoter. (**b**) Western blot analysis of DNAHFVac3.v1 in HEK293T cells 48 h post-transfection using antibodies against 2A peptide, EBOV GP, MARV GP, LASV NP and GAPDH. (**c**) Flow cytometry analysis of DNA-HFVac3.v1 in HEK293T cells 48 h post-transfection using filovirus GP-specific human monoclonal antibodies. (**d**) Expression cassette E-GP.v1 under the p7.5 promoter and M-GP.v1 under control of the short synthetic SSP promoter were inserted into the thymidine kinase (TK) locus J2R, whereas L-NP.v1 was integrated into the Deletion III (Del III) locus under control of the mH5 promoter. DIII F1 (Del III flank 1) and DIII F2 (Del III flank 2) refer to homologous MVA DNA sequences adjacent to the corresponding insertion site of the Del III locus. (**e**) Western blot of HEK293T cells 24 h post-infection (MOI 2) with MVA HFVac3.v1 using antibodies against 2A peptide, EBOV GP, MARV GP, LASV NP, VACV and GAPDH. (**f**) Flow cytometry analysis of MVA-infected cells stained with filovirus GP-specific human monoclonal antibodies. DNA empty, WT MVA and cells only served as controls.

The trivalent DNA vaccine, DNA-HFVac3.v1, encodes RNA- and codon-optimized ORFs for L-NP.v1, M-GP.v1 and E-GP.v1, linked via T2A and P2A sequences to enable translational coupling and co-expression of all three antigens under the control of the human cytomegalovirus (CMV) IE1 promoter (Fig. 2a). Western blot analysis of transiently transfected HEK293T cells confirmed expression and appropriate separation of the antigens via the 2A sites as evidenced by the lack of readthrough proteins (Figs 1b and S3). Of note, our analysis does not provide insights into the exact stoichiometry of the encoded antigens. However, flow cytometry analysis proved the display of E-GP.v1 and M-GP.v1 on the cell surface (Fig. 2c), confirming a decent level of e.g. E-GP.v1 expression, which was encoded by the extreme 3′ part of the expression module.

The recombinant trivalent MVA-HFVac3.v1 vaccine encodes the L-NP.v1 antigen under control of the mH5 promoter in the Del III locus and the E-GP.v1 together with M-GP.v1 in the TK locus, controlled by the p7.5 promoter and the short synthetic promoter (SSP), respectively (Fig. 2d). Western blot analysis of HEK293T cells infected with MVA-HFVac3.v1, compared to MVA WT, confirmed expression of E-GP.v1, M-GP.v1 and L-NP.v1 at the expected molecular weights (Figs 1e and S4). Surface localization of E-GP.v1 and M-GP.v1 was verified by flow cytometry in MVA-HFVac3.v1-infected HEK293T cells (Fig. 2f).

For benchmarking purposes in subsequent immunization studies, we designed, produced and characterized three monovalent DNA- and MVA-vaccine reference constructs encoding EBOV GP (Mayinga/1976) (E-GP.wt), MARV GP (Musoke/1980) (M-GP.wt) and LASV NP (Nigeria/1977) (L-NP.wt), respectively (Fig. S5). Western blot analysis confirmed antigen expression at expected sizes in HEK293T cells after transfection or infection (Fig. S5a–f), and flow cytometry showed surface display of E-GP.wt and M-GP.wt (Fig. S5g–j).

### Evaluation of antibody responses to filovirus GPs following monovalent or trivalent DNA and MVA prime-boost vaccination strategies in BALB/c mice by Luminex assay

BALB/c mice were immunized with three doses of either a combination of three monovalent MVA vaccines expressing the wt antigens E-GP.wt, M-GP.wt and L-NP.wt, or our single trivalent vaccine HFVac3.v1, encoding the corresponding optimized antigen sequence. Vaccination was performed using either a heterologous regimen (prime with DNA, followed by two heterologous MVA boosts) or a homologous regimen (prime with MVA, followed by two homologous MVA boosts) (Fig. 1).

**Fig. 3.**
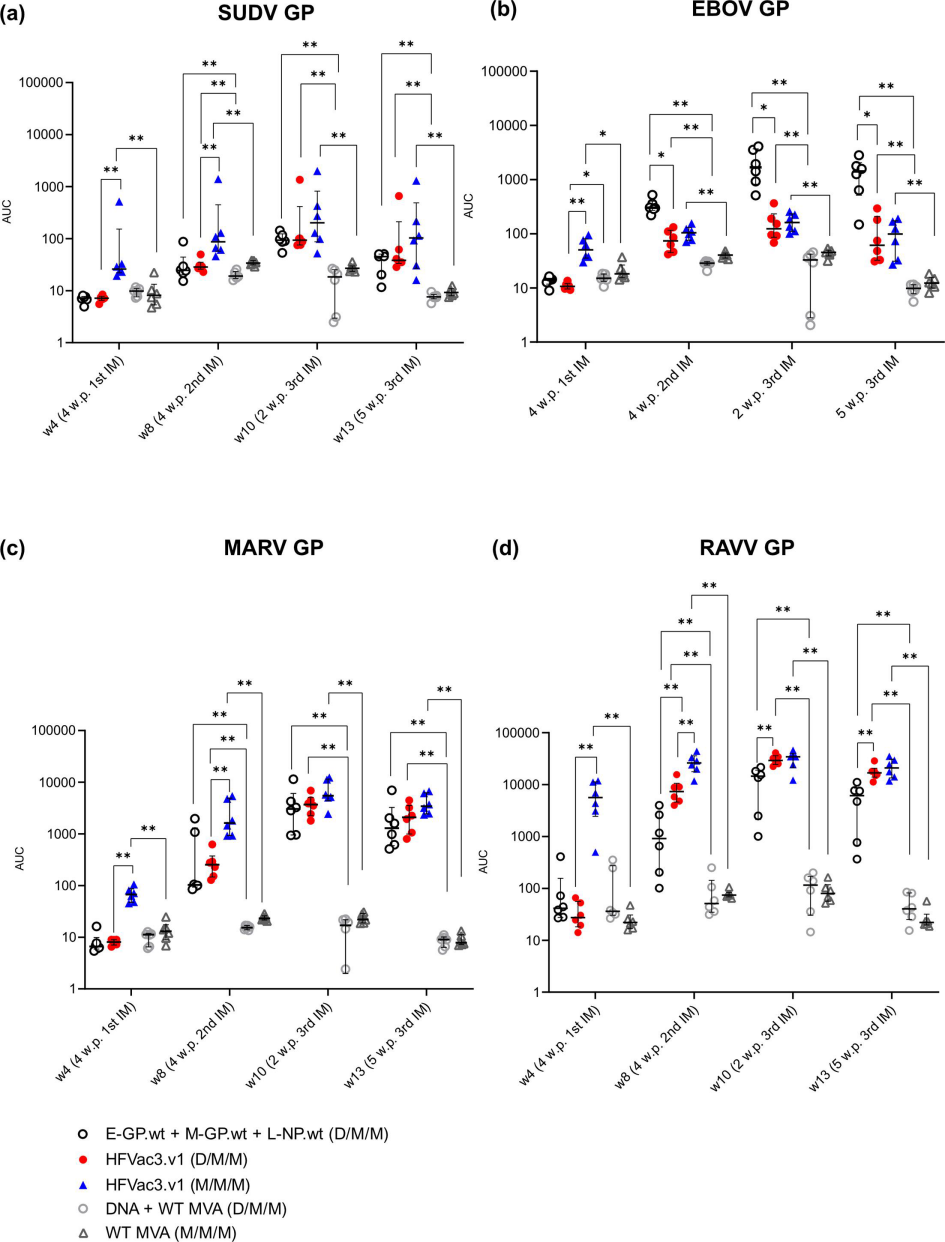
Antibody responses to filovirus GPs elicited by heterologous versus homologous immunizations using monovalent or trivalent DNA and MVA HFVac3.v1 vaccines. BALB/c mice were immunized three times either following a heterologous DNA/MVA/MVA (D/M/M) or homologous MVA/MVA/MVA (M/M/M) prime-boost schedule at weeks 0, 4 and 8 with a mixture of monovalent vaccines (group M1), the trivalent HFVac3.1 vaccine (groups M2 and M3) or empty vector controls (groups M4 and M5). (**a–d**) Binding antibodies measured over time using Luminex assay with the indicated filovirus GPs SUDV GP, EBOV GP, MARV GP and RAVV GP as readout antigens. Median responses and interquartile ranges are indicated, and each symbol represents an individual animal. AUC, area under the curve. Statistical results were calculated using Mann–Whitney U tests with *P<0.05 and **P<0.01. w.p. x IM, weeks post-x immunization.

Four weeks after the first immunization, none of the animals developed detectable antibody levels against the recombinant antigens regardless of whether mice were primed with a mixture of monovalent DNAs encoding wt antigens or the trivalent DNA-HFVac3.v1 vaccine as assessed by Luminex assay (Fig. 3a–d). Following booster immunizations with the mixture of the monovalent MVAs encoding the wt-based antigens, or the single trivalent MVA-HFVac3.v1, antibody responses to the indicated readout antigens (SUDV GP, EBOV GP) developed differently over time. The mixture of wt-based monovalent vaccines (D/M/M, group M1) induced higher titres of EBOV GP-specific antibodies over time (Fig. 3b), reflecting the phylogenetic relationship of the vaccine antigens to the recombinant Orthoebolavirus GPs used in the Luminex assay (Figs S1d, e and S2). SUDV GP-specific titres were generally lower and indistinguishable between the monovalent and trivalent vaccine groups across the different time points. (Fig. 3a). Antibody levels in both immunization groups proved to be durable for at least 5 weeks after the second MVA boost.

There was no significant difference in the levels of binding antibodies against MARV GP following vaccination with the mixture of monovalent vaccines versus the trivalent HFVac3.v1 candidate (Fig. 3c). Binding antibodies induced by the trivalent HFVac3.v1 vaccine showed higher cross-reactivity towards the RAVV GP than those induced by the wt-based antigen (Fig. 3d).

We also compared a homologous (M/M/M, group M3, Fig. 1) versus a heterologous immunization protocol (D/M/M, group M2, Fig. 1) using our trivalent HFVac3.v1 DNA and MVA vaccine candidate.

Binding antibodies against the SUDV GP, EBOV GP, MARV GP and RAVV GP were already detected 4 weeks after the first immunization in mice that were primed with the trivalent MVA-HFVac3.v1. Overall, binding antibodies were significantly lower in D/M/M groups compared to MVA-primed mice, except for EBOV GP-specific antibodies (Fig. 3a–d). In general, antibody levels peaked 2 weeks after the third immunization and were statistically indistinguishable between the DNA- and the MVA-primed groups. Levels of binding antibodies remained high and comparable between the two groups for at least 5 weeks after the third immunization.

In conclusion, the MVA-HFVac3.v1 prime-boost immunization protocol induced robust levels of binding antibodies against SUDV GP, EBOV GP and MARV GP in BALB/c mice.

### Induction of LASV NP-specific binding antibodies and IFN-γ-producing T-cells in BALB/c mice

**Fig. 4.**
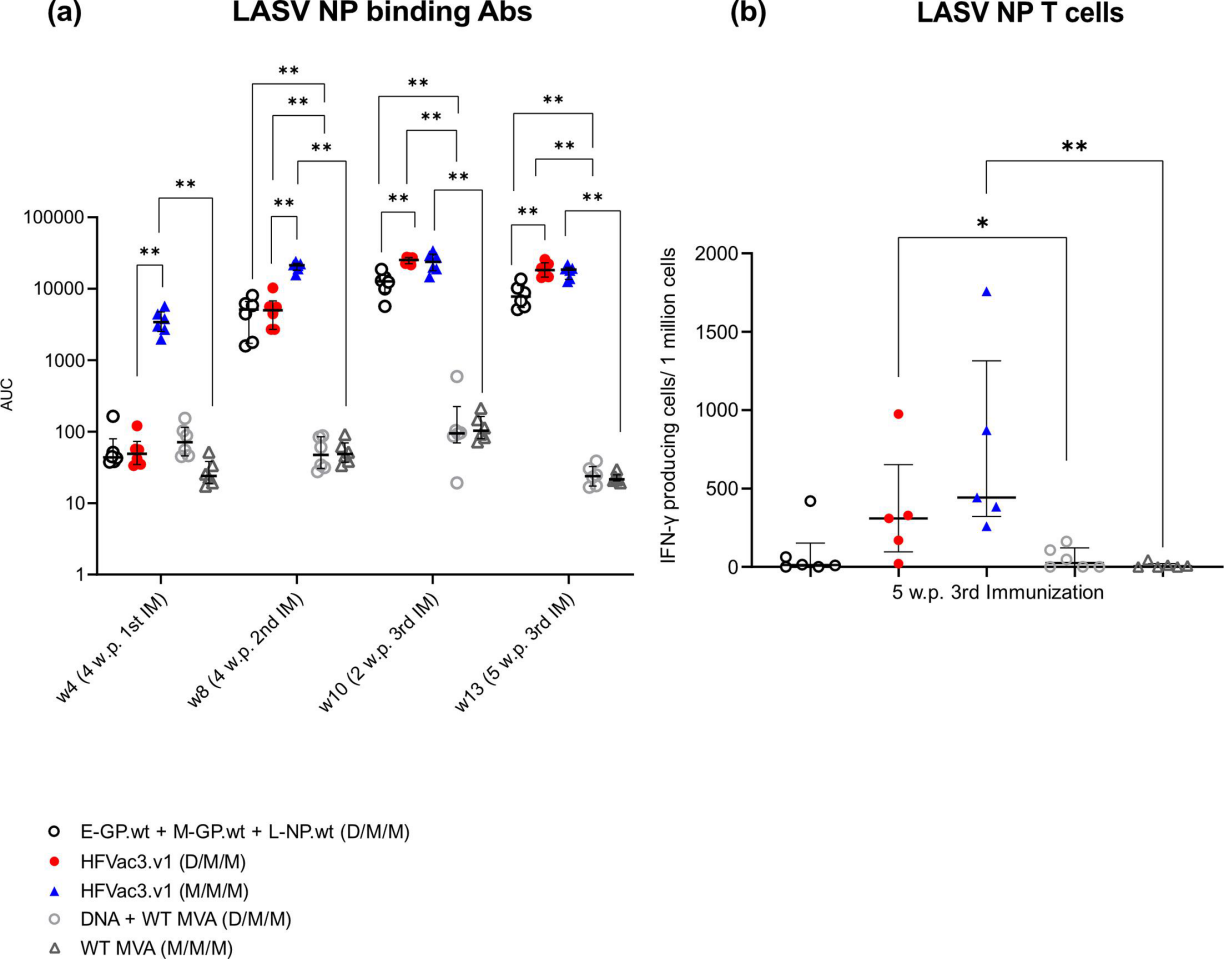
Humoral and cellular immune responses to LASV NP following vaccination using monovalent or trivalent HFVac3.1 vaccines in a heterologous or homologous immunizationM schedule. BALB/c mice were primed with the mixture of monovalent DNA vaccines E-GP.wt, M-GP.wt and L-NP.wt (s.c.) at week 0 and boosted twice at weeks 4 and 8 with the mixture of the corresponding MVAs encoding the identical panel of antigens (D/M/M; group M1). For comparison, mice received a priming immunization either with the trivalent HFVac3.v1 DNA vaccine (D/M/M; group M2) or, alternatively, with the HFVac3.v1 MVA vaccine (M/M/M; group M3) followed by two booster immunizations with MVA-HFVac3.v1. As a control, mice received a priming immunization either with the empty DNA vector or WT MVA, followed by two booster immunizations with WT MVA (groups M4 and M5). (**a**) Binding antibodies measured over time for the LASV NP. (**b**) Cellular immune responses were measured 5 weeks after the third immunization by IFN-γ ELISpot using splenocytes stimulated with a LASV NP peptide pool. Median responses and interquartile ranges are indicated, and each symbol represents an individual animal. AUC, area under the curve. Statistical results were calculated using Mann–Whitney U tests with *P<0.05 and **P<0.01. w.p. x IM, weeks post-x immunization.

In addition to eliciting strong binding antibody responses to filovirus GPs, our HFVac3.v1 vaccine candidates also induced high levels of LASV NP-specific binding antibodies, as measured by Luminex assay. Mice in both heterologous prime-boost groups receiving either the mixture of the monovalent vaccines encoding E-GP.wt, M-GP.wt and L-NP.wt (D/M/M; group M1) or the trivalent HFVac3.v1 vaccine (D/M/M; group M2) developed comparable levels of NP-specific binding antibodies 4 weeks after the first boost with the matched MVA vaccines (Fig. 4a). Two and five weeks after the third immunization with MVA, group M2 mice showed significantly higher levels of NP-specific antibodies than group M1 animals. Noteworthy, similarly to filovirus GP-specific antibody levels, mice that received a priming immunization with MVA-HFVac3.v1 (M/M/M; group M3) responded faster and developed high anti-NP titres already 4 weeks after the priming immunization. Levels of NP-specific antibodies reached their plateau 4 weeks after the first MVA boost. Two weeks after the third immunization with HFVac3.v1, levels of NP-specific antibodies were indistinguishable, regardless of the applied immunization regimen (D/M/M, group M2 versus M/M/M, group M3) (Fig. 4a).

Several reports suggested that LASV NP-specific T-cell responses correlate with protection from LASV infection [48,49]. We therefore also evaluated the number of LASV NP-specific T-cells in murine spleens 5 weeks after the third immunization by ELISpot assay. Our trivalent HFVac3.v1 vaccine candidates (groups M2 and M3) induced IFN-γ secretion in T-cells in response to re-stimulation with the matched LASV NP peptide pool (Fig. 4b). Whereas Lassa NP-specific T-cell responses in the D/M/M group were affected from elevated background in some animals, the M/M/M schedule yielded robust numbers of IFN-γ-positive T-cells. Although there was some tendency towards higher numbers of NP-specific IFN-γ-producing T-cells in group M3 (M/M/M) compared to group M2 mice (D/M/M), this trend did not reach significance (*P*=0.2222).

### Induction of MVA vector-specific antibodies and MPXV-specific binding antibodies

MVA-specific antibodies binding to the vaccinia virus protein extracts were already detected 4 weeks after the priming immunization in group M3 (HFVac3.v1; M/M/M) and the respective control group M5 (MVA-WT, M/M/M) as determined by ELISA. DNA-primed groups M1, M2 and M4 did not develop any MVA-specific antibodies at that time point. Four weeks after the second immunization, MVA-specific antibody levels had already plateaued in groups M3 and M5 and were significantly higher than responses in the DNA-primed groups M1, M2 and M4. Interestingly, MVA-specific antibody titres were higher in the monovalent group M1 (D/M/M) as compared to the trivalent group M2 (MVA HFVac3.v1), probably due to the threefold higher amount of MVA particles delivered in group M1. Two weeks post-third immunization, DNA-primed groups M1, M2 and M4 (D/M/M) were indistinguishable from the MVA-primed groups M3 and M5 (M/M/M), indicating that two MVA exposures lead to maximal MVA-specific-antibody levels (Fig. S6).

We also analysed binding antibodies using a Luminex assay targeting surface proteins from both mature virions (MV; M1 and H3) and extracellular virions (EV; A35 and B6) of MPXV. These antigens have been previously associated with neutralization activity and protection against lethal clade I MPXV infection in NHPs [50,51]. Our trivalent HFVac3.v1 vaccine induced high levels of binding antibodies against all four different MPXV proteins regardless of whether animals were vaccinated with the heterologous or homologous immunization schedule. Neither the transgene expression nor a DNA prime negatively impacted the level of MPXV-specific binding antibody responses in mice (Fig. S7).

### Induction of binding and neutralizing antibodies following vaccination with the trivalent HFVac3.v1 vaccine in guinea pigs

**Fig. 5.**
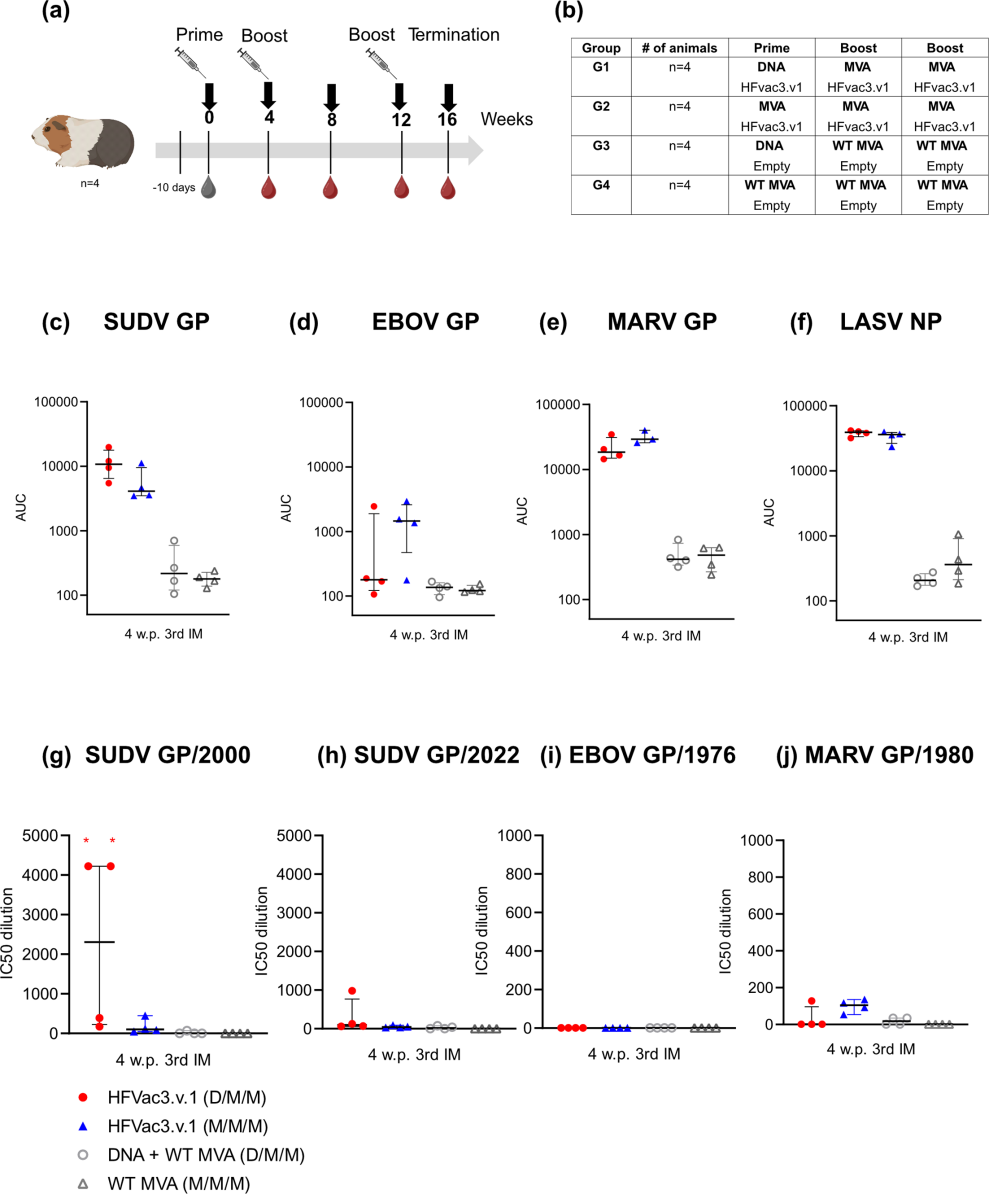
Binding and neutralizing antibodies raised in guinea pigs against different filovirus-derived GPs and LASV NP. (**a**) Hartley guinea pigs (*n*=4) were immunized on day 0 with either HFVac3.v1 DNA via the intradermal route (i.d.) or HFVac3.v1 MVA (i.m.) followed by two MVA immunizations at weeks 4 and 12 (timeline and experimental design created with BioRender.com). The study was terminated at week 15. (**b**) Hartley guinea pigs were vaccinated with the trivalent HFVac3.1 vaccines either in a heterologous (D/M/M; group G1) or homologous (M/M/M; group G2) immunization regimen. As controls, empty DNA or WT MVA (groups G3 and G4) were used, respectively. (**c–f**) Binding antibodies were analysed from the terminal bleeds using Luminex for the indicated filovirus GPs from SUDV, EBOV, MARV and the LASV NP. (**g–j**) Neutralizing antibodies against SUDV/Gulu/2000, SUDV/Uganda/2022, EBOV/Mayinga/1976 and MARV/Musoke/1980 were examined 3 weeks after the last MVA immunization using lentiviral vectors pseudotyped with the mentioned GPs. Calculated IC50 values for each animal are shown against (**g**) SUDV 2000 PV, (**h**) SUDV 2022 PV, (**i**) EBOV PV and (**j**) MARV PV. Each symbol represents one animal. In panel (**g**), two animals out of four animals in total are depicted with a red star, indicating that these two animals had higher than maximum IC50 dilution that could be derived from this experimental setup (the respective sera were not fully titrated in the neutralization assay). Median responses and interquartile ranges are indicated, and each symbol represents an individual animal. IM, immunization. AUC, area under the curve. w.p. x IM, weeks post-x immunization.

As Hartley guinea pigs are generally accepted as a relevant animal model for challenge studies with EBOV, MARV and LASV, we determined the capacity of the DNA- and MVA-HFVac3.v1 vaccine to induce binding and neutralizing antibodies in an exploratory guinea pig pilot study. For that purpose, four female Hartley guinea pigs per group were vaccinated with the trivalent HFVac3.v1 vaccine at weeks 0, 4 and 12 either using a heterologous (group G1, D/M/M) or homologous (G2, M/M/M) immunization protocol. Empty DNA or MVA vaccine constructs served as negative controls (G3 and G4) (Fig. 5a, b).

Luminex analysis of the terminal bleed revealed that the trivalent vaccine induced high levels of binding antibodies against SUDV GP, MARV GP and LASV NP (Fig. 5c, e and f). (Fig. S8).

Levels of EBOV GP-specific binding antibodies were lower in all animals of G1 and G2 as compared to SUDV GP-specific antibodies. Except for EBOV GP-specific antibodies, titres in G2 (M/M/M) tended to be higher as compared to G1 (D/M/M). Levels of binding antibodies against all tested filovirus GPs and LASV NP were similar between the heterologous and the homologous immunization regimen, although no statistics were calculated due to the low number of animals. Whereas mice did benefit from an MVA priming immunization compared to a DNA prime in terms of early GP-specific antibody kinetics, this advantage was not observed in the small number of guinea pigs at earlier time points between the M/M/M and D/M/M groups.

Although previous studies suggest that neutralizing antibodies are not required to confer protection [52,53], we also determined neutralizing antibodies using a lentiviral pseudotype assay covering a range of different filoviruses. We observed that our trivalent vaccine induced moderate to high levels of SUDV-specific neutralizing antibodies in some guinea pigs (2/4 for SUDV GP/2000 and 1/4 for SUDV GP/2022) (Fig. 5g, h), whereas other animals only showed low levels or no neutralizing antibodies. For EBOV GP (EBOV/1976), no neutralizing antibodies were detected. Guinea pigs immunized with M/M/M showed low levels of neutralizing antibodies against MARV PV (MARV/1980) compared to the controls (Figs 5i, j and S9).

We also assessed MPXV binding antibodies in Hartley guinea pigs by Luminex assay. Our trivalent HFVac3.v1 MVA vaccine induced high levels of binding antibodies against MPXV proteins A35, M1, H3 and B6. Regarding M1, DNA-primed guinea pigs (D/M/M) developed lower levels of binding antibodies compared to the animals vaccinated with the homologous (M/M/M) immunization protocol. A similar trend was observed for all other MPXV antigens tested (Fig. S10). Furthermore, contrasting our data in BALB/c mice, HFVac3.v1 transgene expression seems to mitigate the development of MPXV-specific antibodies. This becomes most obvious when DNA is used for priming immunizations (B6-, H3- and M1-specific antibodies), and guinea pigs were only exposed twice to MVA. This is being partially compensated after three MVA injections, but for M1 (and by trend also for B6), the difference in antibody levels remains.

In summary, we demonstrated that our trivalent HFVac3.v1 vaccine candidate induces binding antibodies against all three recombinant antigens, neutralizing antibodies against SUDV and T-cell responses against LASV NP along with binding antibodies against cross-reactive MPXV antigens in two animal species.

## Discussion

A cost-effective, multivalent vaccine that effectively prevents infection or severe disease is highly desirable to reduce mortality and morbidity associated with VHFs. Here, we present a novel trivalent MVA-based vaccine candidate HFVac3.v1 expressing three digitally optimized antigen sequences for Orthoebolavirus GP, Orthomarburgvirus GP and Lassa virus NP in a single vector. This trivalent MVA HFVac3.1 vaccine was used alone or in a heterologous prime-boost regimen with a matching trivalent DNA vaccine for priming. For benchmarking, we decided to control our trivalent HFVac3.v1 vaccine against a mixture of monovalent MVAs and DNA vaccines encoding antigens that were used in approved vaccines (E-GP.wt; Ervebo^®^, Zabdeno/MVA-BN Filo) or vaccine candidates that were extensively tested in clinical studies (M-GP.wt and L-NP.wt), respectively. We refrained from establishing a trivalent MVA encoding the wt antigens primarily because we felt that the mixture of the monovalent vaccines expressing the antigens independently from each other may be the more rigorous control.

Our study demonstrated in two different animal species – BALB/c mice and Hartley guinea pigs – that our trivalent HFVac3.v1 vaccine approach induces robust antibody responses to SUDV GP, MARV GP and LASV NP. ELISpot analysis further confirmed LASV NP-specific T-cells in mice. The capacity of MVA to integrate several transgenes into one single vector allows potential tetravalent protection against SUDV, MARV and LASV and the closely related MPXV. Importantly, MVA-HFVac3.v1 triggered high levels of MPXV-specific binding antibodies (A35, M1, H3 and B6), which have shown protection in NHPs [25,54], supporting further studies to evaluate MPXV protection in appropriate animal models.

When we compared our trivalent HFVac3.v1 vaccine (D/M/M) encoding the set of optimized antigens to the mixture of monovalent vaccines encoding wt antigens in BALB/c mice, titres of binding antibodies to the selected antigens were comparable except for EBOV GP, where the mixture of monovalent vaccines yielded overall higher titres. Due to our study design, we cannot conclude whether this is due to the exact match of the EBOV GP antigen used in the Luminex assay and the vaccine-encoded E-GP.wt, or a consequence of the vaccine composition (mixture of monovalent vaccines versus trivalent vaccine).

Our HFVac3.1 vaccine preferentially induced SUDV GP-specific antibodies, which tended to cross-react with recombinant GPs derived from two SUDV strains (SUDV/2000 and SUDV/2022), though neutralization titres varied depending on the used lentiviral pseudotype. EBOV GP binding antibodies were rather low, and no cross-neutralization was observed in guinea pigs. Due to the small group sizes of guinea pigs, no conclusion can be made whether DNA priming immunization adds to the outcome of antibody responses. The question remains whether further improved computational antigen design could help to bridge the antigenic gap between EBOV and SUDV, and possibly other Orthoebolavirus species including Bundibugyo virus, Reston virus or Taï Forest virus. One strategy could be to focus more on closely related variants (as shown in this study for SUDV) and to combine GPs from different contemporaneous Orthoebolavirus strains into one vaccine. Such combined vaccine approaches have already been proven protective in NHPs against EBOV, SUDV and MARV, using either a two-dose heterologous regimen (Ad26.Filo followed by MVA-BN-Filo) [55] or a combined recombinant VSV-based vaccine, which conferred protection against EBOV, SUDV, MARV and LASV [56]. Interestingly, recent findings showed that a bivalent adenoviral vaccine targeting both EBOV and SUDV GP did not protect NHPs from a lethal SUDV challenge [57] but was immunogenic and well tolerated in humans [58]. To what extent differences in study outcomes can be attributed to the employed animal models, vaccine vectors, immunization schedules, routes or other factors is currently unclear.

Because of the high mortality rate of MARV and the lack of an approved vaccine, we included MARV GP in our trivalent vaccine design. While we observed high levels of MARV and RAVV GP-specific antibodies, no neutralizing antibodies were detected. Previous studies demonstrated vaccine-induced protection even without neutralizing antibodies, and MARV GP-specific binding antibodies and Fc effector functions may play a protective role [53,59].

Currently approved vaccines target EBOV GP, and Ervebo^®^ has shown only limited cross-protection against SUDV [60]. No vaccines have been approved for SUDV or MARV to date [61], and LASV is not included as a target antigen in these vaccines. While correlates of protection against LASV remain unclear [48,62], studies [63,65] and clinical evidence [49,66, 67] suggest that protection from LASV infection can occur without neutralizing antibodies, with binding antibodies and/or CD8+ T-cell responses playing a critical role.

In the LCMV arenavirus mouse model, NP-specific T-cell responses were crucial for controlling infection. Recently, a new mechanism of immune synergy between NP-specific antibodies and T-cells was identified, where cellular uptake of NP-antibody immune complexes, followed by Fc-receptor-mediated targeting of NP to the E3 ubiquitin ligase TRIM21, enhanced T-cell responses [68]. This led us to include LASV NP as a T-cell antigen in our trivalent VHF vaccine. As designed, our HFVac3.v1 vaccine induced high levels of LASV NP-specific binding antibodies in two animal models. NP-specific IFN-γ-specific T-cell responses were particularly pronounced in the M/M/M group and were, by trend, higher as compared to the D/M/M group. Future analysis should look more carefully into the composition of T-cell subpopulations (CD3, CD4 and CD8) including – but not limited to – polyfunctional T-cells by looking at effector cytokines (IFN-γ, IL-2 and TNF-α), degranulation (CD107a), memory T-cells (e.g. central memory, effector memory or tissue-resident memory) or T follicular helper cell responses (e.g. CXCR5, PD-1 and ICOS).

In BALB/c mice, the homologous M/M/M regimen of HFVac3.v1 outperformed the heterologous D/M/M regimen, with faster development of EBOV GP, SUDV GP, MARV GP and LASV NP-specific antibodies after a single MVA priming. This suggests that MVA-HFVac3.v1 may be suitable for both acute outbreak and prophylactic use. A homologous vaccination schedule would simplify vaccine production and logistics of vaccination campaigns, as only one component would need to be produced to GMP quality.

As Hartley guinea pigs are considered a relevant model for studying haemorrhagic fever viruses [69,70], we conducted a preliminary pilot study using our trivalent MVA and DNA-based HFVac3.v1 vaccine candidates to prepare for challenge studies. Unlike BALB/c mice, in guinea pigs, there was no difference between the heterologous D/M/M and homologous M/M/M regimens in terms of antibody kinetics or binding antibody levels. This discrepancy could be due to differences in the nature of antigen(s), expression kinetics, T-cell epitope potency or the interplay of antigens with DNA and MVA delivery platforms. Additionally, the small number of animals in our guinea pig study did not allow for formal statistical analysis. Nevertheless, the immunological data provide valuable information for determining the appropriate sample size required to achieve sufficient statistical power in guinea pig efficacy studies. Due to limited reagents for guinea pigs, we could not assess T-cell responses or Fc-mediated functions, which are thought to play a role in protection. Furthermore, no NIBSC international standards are available for guinea pig immune sera for readout standardization. Ongoing, properly powered challenge studies in Hartley guinea pigs using guinea pig-adapted SUDV, MARV and LASV live viruses are specifically designed to strengthen the link between vaccine efficacy and immune effector functions. Notably, each model to study haemorrhagic fever viruses – such as *Ifnar^-/-^* mice [71], Hartley guinea pigs [72], ferrets [73] and NHPs [52,57] – has its own strengths and limitations, but they collectively provide complementary insights. Given the variability in predictive power across different animal models, NHP studies are especially valuable due to their higher translational relevance. Integrating cross-model immunogenicity data alongside expanded NHP studies will be key for improving our understanding of protective responses in humans [69,70, 74].

MPXV-specific neutralizing antibodies were demonstrated to confer protection from MPXV infection in NHPs [50,75, 76] and in humans [26]. Noteworthy, following vaccination with MVA-HFVac3.v1, mice and guinea pigs developed high levels of binding antibodies against a selected panel of neutralization-sensitive MPXV proteins, even after one immunization. In guinea pigs, we observed a clear trend towards higher antibody titres after three immunizations with MVA HFVac3.v1 compared to the heterologous D/M/M immunization.

In conclusion, the trivalent HFVac3.v1 vaccine demonstrates the potential to elicit cross-reactive binding antibodies against various filoviruses, high titres of LASV NP-specific antibodies in both tested animal species, respectively, and NP-specific T-cell responses in mice. Additionally, MVA-specific antibodies showed broad cross-reactivity to neutralization-sensitive MPXV proteins. While post-outbreak EBOV vaccines are available, the continued threat from other filoviruses, annual LASV epidemics and rising MPXV cases emphasizes the need for an MVA-based trivalent vaccine for high-risk populations in SSA and justifies further development of HFVac3.v1 for clinical testing.

## Supplementary material

10.1099/jgv.0.002157 Uncited Supplementary Material 1.

## Abbreviations

AUC: area under the curve
CDS: coding sequence
CMV: cytomegalovirus
Del III: Deletion III
DMEM: Dulbecco’s MEM
EBOV: Ebola virus
GP: glycoprotein
i.d.: intradermal route
i.m.: intramuscular route
LASV: Lassa virus
LF: Lassa fever
MARV: Marburg virus
MFI: mean fluorescence intensity
MPXV: mpox virus
MVA: Modified Vaccinia Ankara
NHPs: non-human primates
NP: nucleoprotein
P2A: porcine teschovirus-1 2A
Pen/Strep: penicillin/streptomycin
RAVV: Ravn virus
ROC: receiver operating characteristic
s.c.: subcutaneous route
SSA: Sub-Saharan Africa
SSP: short synthetic promoter
SUDV: Sudan virus
T2A: thosea asigna virus 2A
TAFV: Taï Forest virus
TK: thymidine kinase
VHFs: viral haemorrhagic fevers
VSV: vesicular stomatitis virus
