# Supplementary material for: Immunogenicity of a trivalent haemorrhagic fever vaccine candidate against Sudan virus, Marburg virus and Lassa virus in an mpox vaccine
Source: J Gen Virol. 2025 Oct 6;106(10):002157. doi: 10.1099/jgv.0.002157 (PMC12500382; doi:10.1099/jgv.0.002157)
Supplement: Uncited Supplementary Material 1. [file jgv-106-02157-s001.pdf]

Supplementary Material

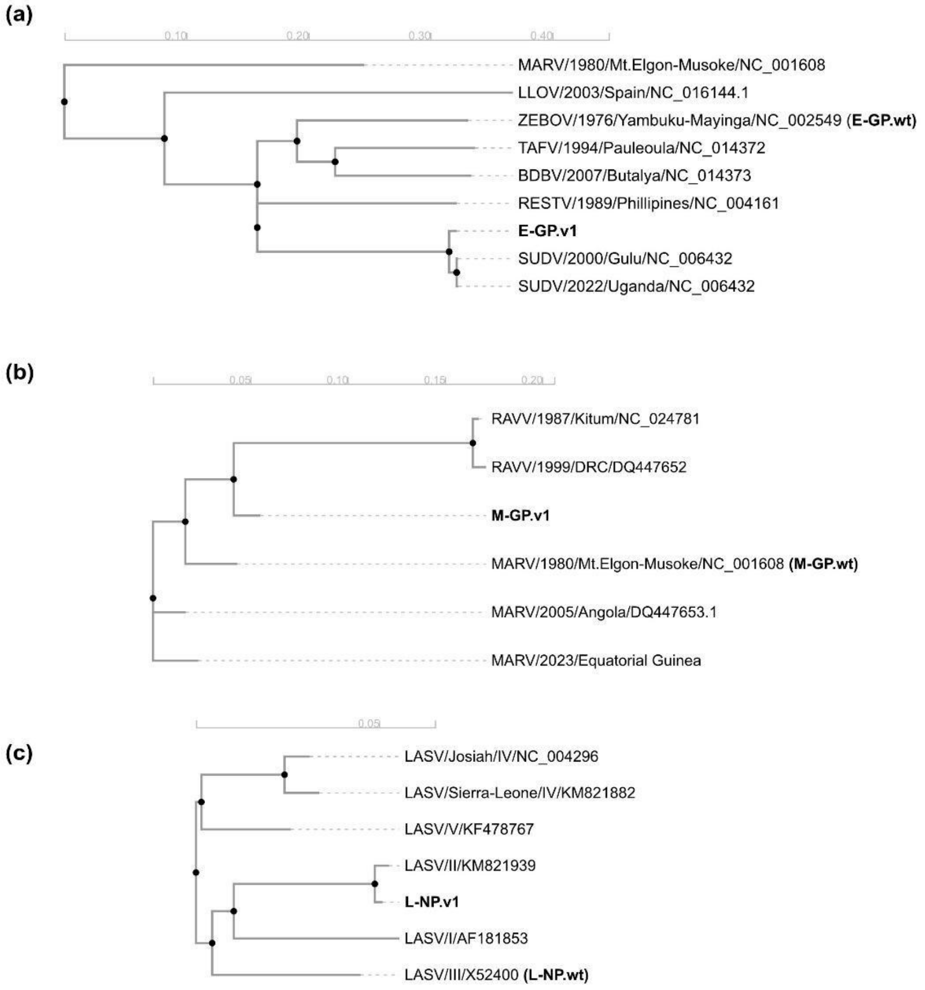

(d)

Luminex readout	Recombinant protein
**SUDV GP**	SUDV/2000/Gulu
**ZEBOV GP**	ZEBOV/1976/Yambuku-Mayinga
**MARV GP**	MARV/1980/Mt.Elgon-Musoke
**RAVV GP**	RAVV/1987/Kitum
**LASV NP**	LASV/III

(e)

Neutralization readout	Lentiviral pseudtypes
**SUDV PV**	SUDV/2000/Gulu; SUDV/2022/Uganda
**ZEBOV PV**	ZEBOV/1976/Yambuku-Mayinga
**MARV PV**	MARV/1980/Mt.Elgon-Musoke

Supplementary figure S1. Phylogenetic analysis of designed immunogens.

Glycoprotein sequences derived from the most clinically relevant and outbreak-associated Orthoebolaviruses and Orthomarburgviruses and the NP amino acid sequences of different Lassa fever virus isolates were extracted from the NCBI database. Multiple sequence alignments were performed using Clustal W and phylogenetic trees for Orthoebolavirus GP sequences (a) Orthomarburgvirus GP sequences (b) and Lassa virus NP sequences (c) were calculated using MUSCLE. The immunogens used for the monovalent vaccines (E-GP.wt, M-GP.wt, L-NP.wt) and trivalent HFVac3.1 vaccines (E-GP.v1, M-GP.v1, L-NP.v1) are indicated in the phylogenetic tree using bold letters. An overview of recombinant proteins used in the Luminex assay (d) and lentiviral pseudotypes used for neutralization assays (d) is depicted.

(a)

MARV/1980/Mt.Elgon-Musoke	100	29,78	28,96	28,53	28,86	29,66	30,28	30,28
ZEBOV/1976/Yambuku-Mayinga	29,78	100	65,16	65,77	59,37	56,84	56,69	56,69
TAFV/1994/Pauleoula	28,96	65,16	100	73,2	58,81	56,27	56,27	56,12
BDBV/2007/Butalya	28,53	65,77	73,2	100	59,88	56,37	56,37	56,37
RESTV/1989/Phillipines	28,86	59,37	58,81	59,88	100	63,13	62,67	62,67
E-GP.v1	29,66	56,84	56,27	56,37	63,13	100	98,52	98,22
SUDV/2000/Gulu	30,28	56,69	56,27	56,37	62,67	98,52	100	99,7
SUDV/2022/Uganda	30,28	56,69	56,12	56,37	62,67	98,22	99,7	100

(b)

RAVV/1987/Kitum	100	98,83	83,26	78,12	77,83	77,53
RAVV/1999/DRC	98,83	100	82,67	77,68	77,39	77,09
M-GP.v1	83,26	82,67	100	92,66	91,19	90,16
MARV/1980/Mt.Elgon-Musoke	78,12	77,68	92,66	100	92,8	91,92
MARV/2005/Angola	77,83	77,39	91,19	92,8	100	95,15
MARV/2023/Equatorial Guinea	77,53	77,09	90,16	91.92	95,15	100

(c)

LASV/I	100	89,81	89,81	88,93	90,16	90,51	89,98
LASV/II	89,81	100	99,3	89,81	90,69	89,98	89,63
L-NP.v1	89,81	99,3	100	90,16	90,69	90,33	89,98
LASV/III	88,93	89,81	90,16	100	91.92	90,86	90,51
LASV/V	90,16	90,69	90,69	91,92	100	93,5	93,5
LASV/IV/Josiah	90,51	89,98	90,33	90,86	93,5	100	98,07
LASV/IV/Sierra-Leone	89,98	89,63	89,98	90,51	93,5	98,07	100

Supplementary figure S2. Percent identity matrix analysis of vaccine antigens.

The precent identity matrix analysis of GP sequences derived from the from the most clinically relevant and outbreak-associated Orthoebolaviruses and Orthomarburgviruses and the NP amino acid sequences of different Lassa fever virus isolates and the designed antigens E-GP.v1 (a), M-GP.v1 (b), L-NP.v1 (c) was created by Clustal2.1.

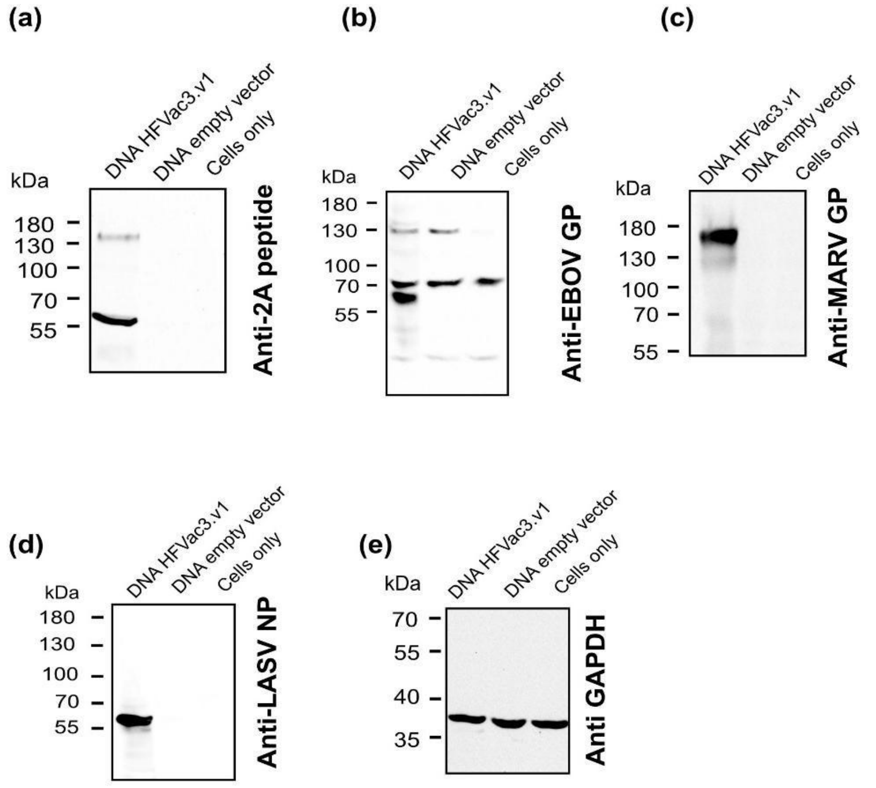

Supplementary figure S3. Antigen expression of trivalent DNA-based HFVac3.v1 vaccine candidate.

HEK293T cells were transfected with DNA-HFVac3.v1 and cells were harvested at 48 h after transfection. Antigen expression of DNA-HFVac3.v1 was analysed by Western blot.

Membranes were stained with antigen-specific antibodies against (a) 2A-peptide (monoclonal) (calculated and expected molecular weight ~63 kDa for LASV NP and ~ 130 kDa for MARV GP) (b) EBOV GP (polyclonal) (calculated and expected molecular weight ~ 58 kDa (c) MARV GP (monoclonal) (calculated and expected molecular weight ~ 130 kDa) (d) LASV NP (monoclonal) (calculated and expected molecular weight ~63 kDa (e) GAPDH (monoclonal) (calculated and expected molecular weight ~37 kDa). As negative control, cell lysates transfected with DNA empty vector and cells only were used. GAPDH was used as a loading control. The PageRuler™ Prestained Protein Ladder, used as standard for calculating protein sizes in kDa, is shown on the left. Additional bands seen in Western blot stained with the polyclonal EBOV GP antibody represent non-specific background signals of the antibody to endogenous cellular proteins.

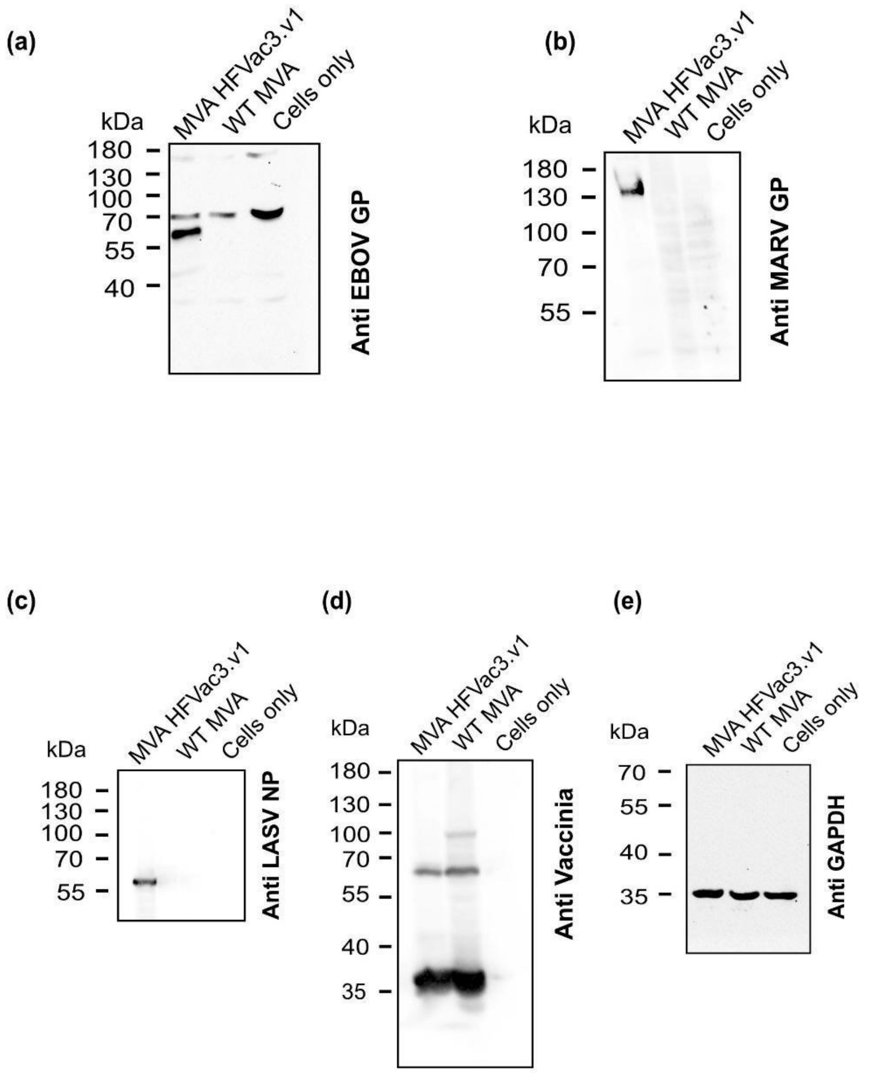

Supplementary figure S4. Antigen expression of trivalent MVA-based HFVac3.v1 vaccine candidate.

Membranes were stained with antigen-specific antibodies against (a) EBOV GP (polyclonal) (calculated and expected molecular weight ~ 58 kDa (b) MARV GP (monoclonal) (calculated and expected molecular weight ~ 130 kDa) (c) LASV NP (monoclonal) (calculated and expected molecular weight ~63 kDa (d) Vaccinia proteins (polyclonal) calculated and expected molecular weight ~35 kDa (e) GAPDH (monoclonal) (calculated and expected molecular weight ~37 kDa). As negative control, cell lysates infected with WT MVA and cells only were used. Vaccinia was used as a MVA infection control, whereas GAPDH was used as a loading control. The PageRuler™ Prestained Protein Ladder, used as standard for calculating protein sizes in kDa, is shown on the left.

Additional bands seen in Western blot stained with the polyclonal EBOV GP antibody represent non-specific background signals of the antibody to endogenous cellular proteins.

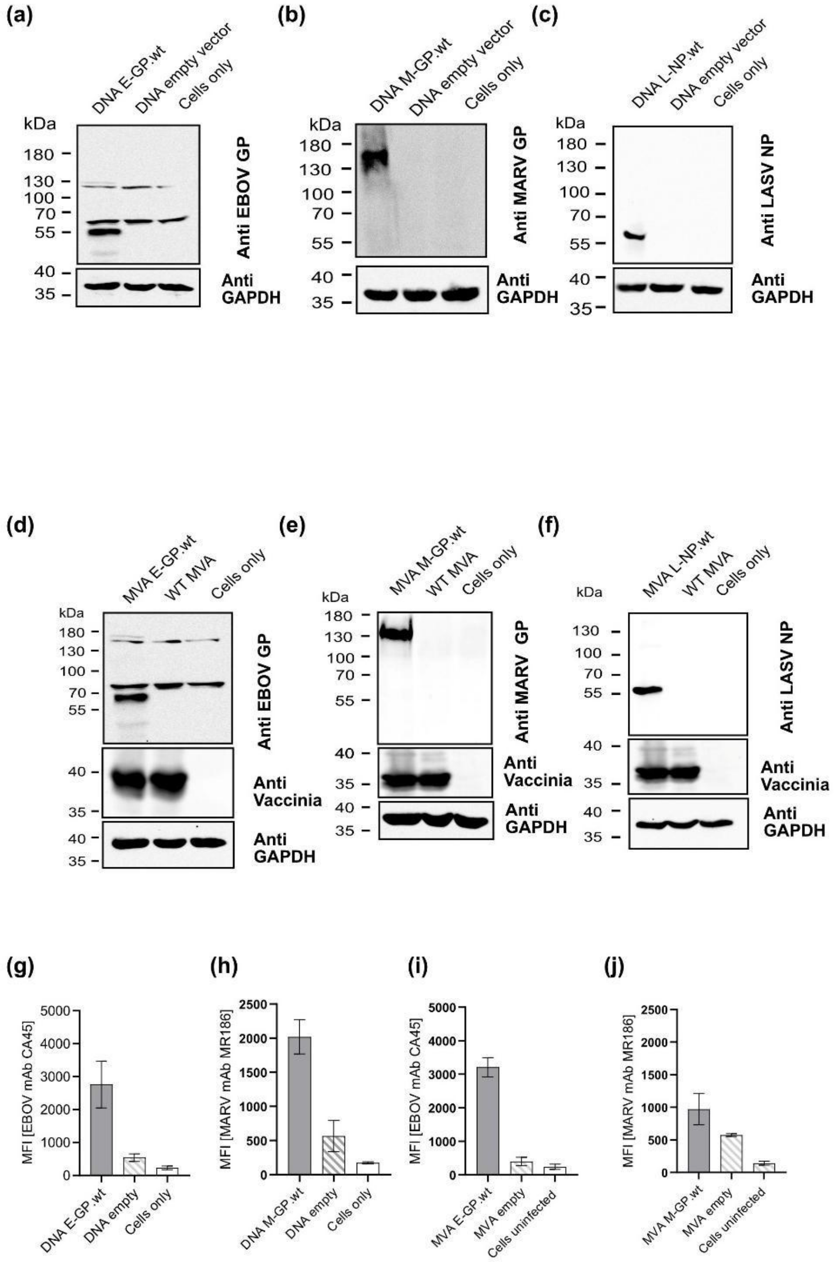

Supplementary figure S5. Antigen expression of monovalent DNA- and MVA-based reference constructs encoding wildtype antigens.

HEK293T cells were transfected with the monovalent DNAs, respectively, and cells were harvested at 48 h after transfection. For testing the expression of the monovalent MVAs by Western blot, HEK293T cells were infected at a MOI of 2 and cells were harvested after 24 h. Membranes were stained with antigen-specific antibodies against (a,d) EBOV GP (polyclonal) (calculated and expected molecular weight ~ 58 kDa (b,e) MARV GP (monoclonal) (calculated and expected molecular weight ~ 130 kDa) (c,f) LASV NP (monoclonal) (calculated and expected molecular weight ~63 kDa (d) Vaccinia proteins (polyclonal) calculated and expected molecular weight ~35 kDa (e) GAPDH (monoclonal) (calculated and expected molecular weight ~37 kDa). As negative control, cell lysates transfected with DNA empty vector/infected with WT MVA and cells only were used. Vaccinia was used as a MVA infection control, whereas GAPDH was used as a loading control. The PageRuler™ Prestained Protein Ladder, used as standard for calculating protein sizes in kDa, is shown on the left. Additional bands seen in Western blot stained with the polyclonal EBOV GP antibody represent non-specific background signals of the antibody to endogenous cellular proteins. Cell surface expression of M-GP.wt and E-GP.wt following transfection of the DNA vaccines **(g,h)** or infection with the MVA vaccines **(i,j)** was analysed by flow cytometry.

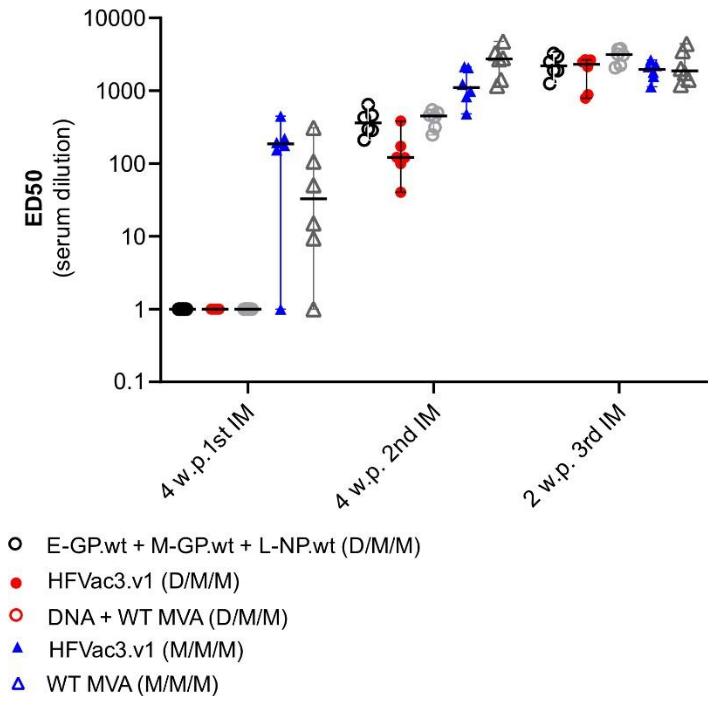

Supplementary Figure S6. Kinetics of MVA-specific binding antibodies in BALB/c mice.

BALB/c (n=6 per group) were primed with a mixture of monovalent DNA vaccines (s.c.) at week 0 and boosted twice at weeks 4 and 8 with the mixture of monovalent MVA vaccines). For comparison, mice received a priming immunization either with the trivalent HFVac3.v1 DNA vaccine or, alternatively, with the HFVac3.1 MVA vaccine, followed by two booster immunizations with the HFVac3.1 (groups M2 and M3). As control, mice received a priming immunization either with the empty DNA vector or WT-MVA, followed by 2 booster immunizations with WT-MVA groups M4 and M5). MVA-specific binding antibodies against MVA are shown four weeks after the first and second and two weeks after the 3rd immunization. Titers are shown as ED50 (Serum dilution) values. Median responses and interquartile ranges are indicated, and each symbol represents an individual animal. w.p. x IM – weeks post x immunization.

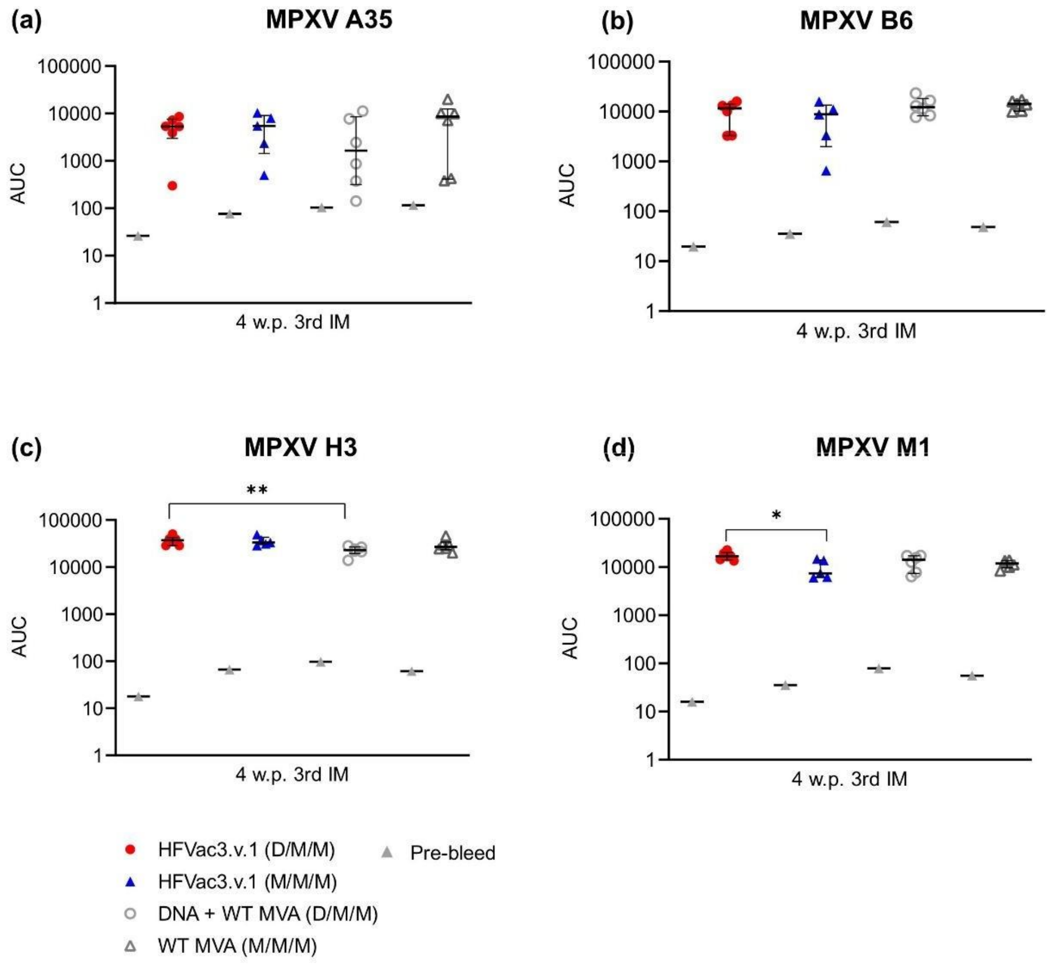

Supplementary Figure S7. Binding antibody responses to the indicated MPXV proteins elicited by heterologous versus homologous immunizations with the trivalent HFVac3.1 vaccines in BALB/c mice.

BALB/c were immunized three times following a heterologous DNA/MVA/MVA (group M2; D/M/M) or homologous MVA/MVA/MVA (group 3; M/M/M) prime-boost schedule at week 0, 4 and 8 with the trivalent HFVac3.1 vaccine (groups M2, M3) or empty vector controls (groups M4, M5). (**a-d)** Binding antibodies were analysed from the terminal bleeds using Luminex for the indicated MPXV proteins of mature and extracellular virions. Median responses and interquartile ranges are indicated, and each symbol represents an individual animal. Pre-bleeds of each group are depicted as grey triangles. AUC: Area under the curve. Statistical results were calculated using Mann-Whitney U-tests with * - $p < 0.05$; **- $p < 0.01$. w.p. x IM – weeks post x immunization.

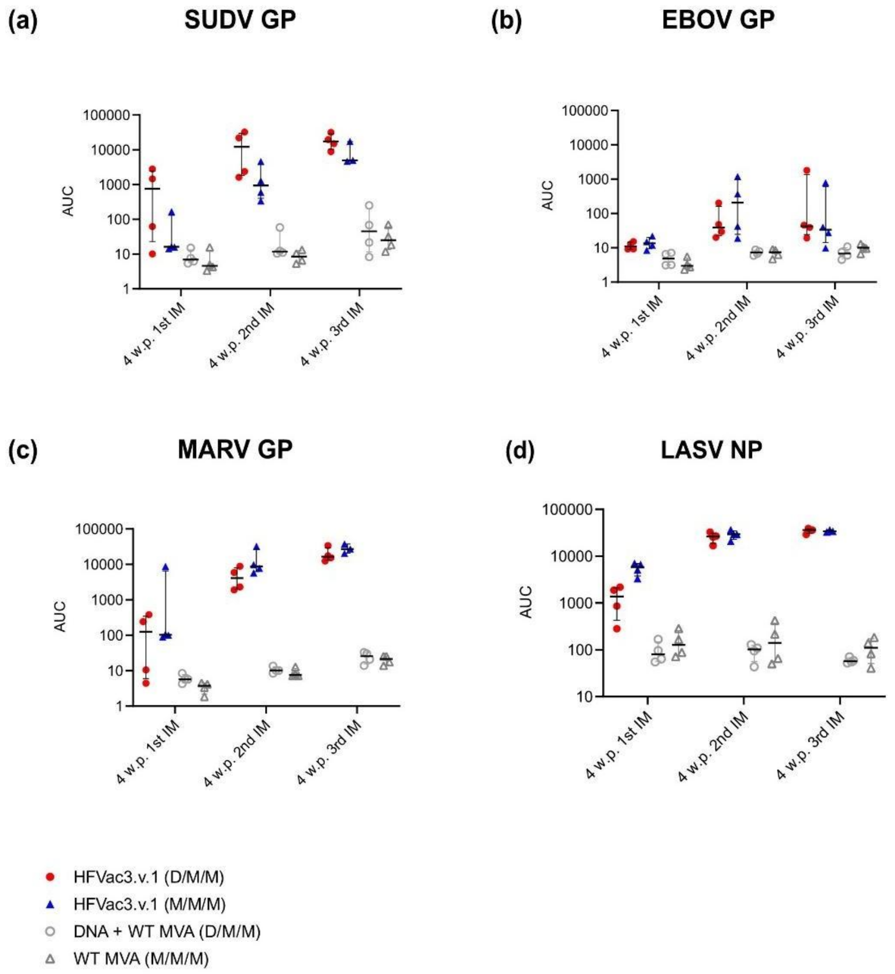

Supplementary Figure S8. Binding antibodies kinetics in Hartley guinea pigs.

Supplementary to Figure 6. Female Hartley guinea pigs (n=4 per group) were primed with HFVac3.v1 DNA vaccine (s.c.) or the HFVac3.v1 MVA vaccine (i.m.) at week 0 and boosted twice at week 4 and 8 with the HFVac3.v1 MVA vaccine (i.m.). For control, guinea pigs were primed with either with the empty DNA vector or WT-MVA, followed by 2 booster immunizations with WT-MVA. Binding antibodies against (a) SUDV GP, (b) EBOV GP, (c) MARV GP and (d) LASV NP are shown four weeks after the first, second and 3rd immunization. Titres are shown as area under the curve (AUC) of antibody binding curves measured with Luminex using recombinant proteins. Median responses and interquartile ranges are indicated, and each symbol represents an individual animal Statistical results were calculated using Mann-Whitney U-tests with * - $p < 0.05$; **- $p < 0.01$. w.p. x IM – weeks post x immunization

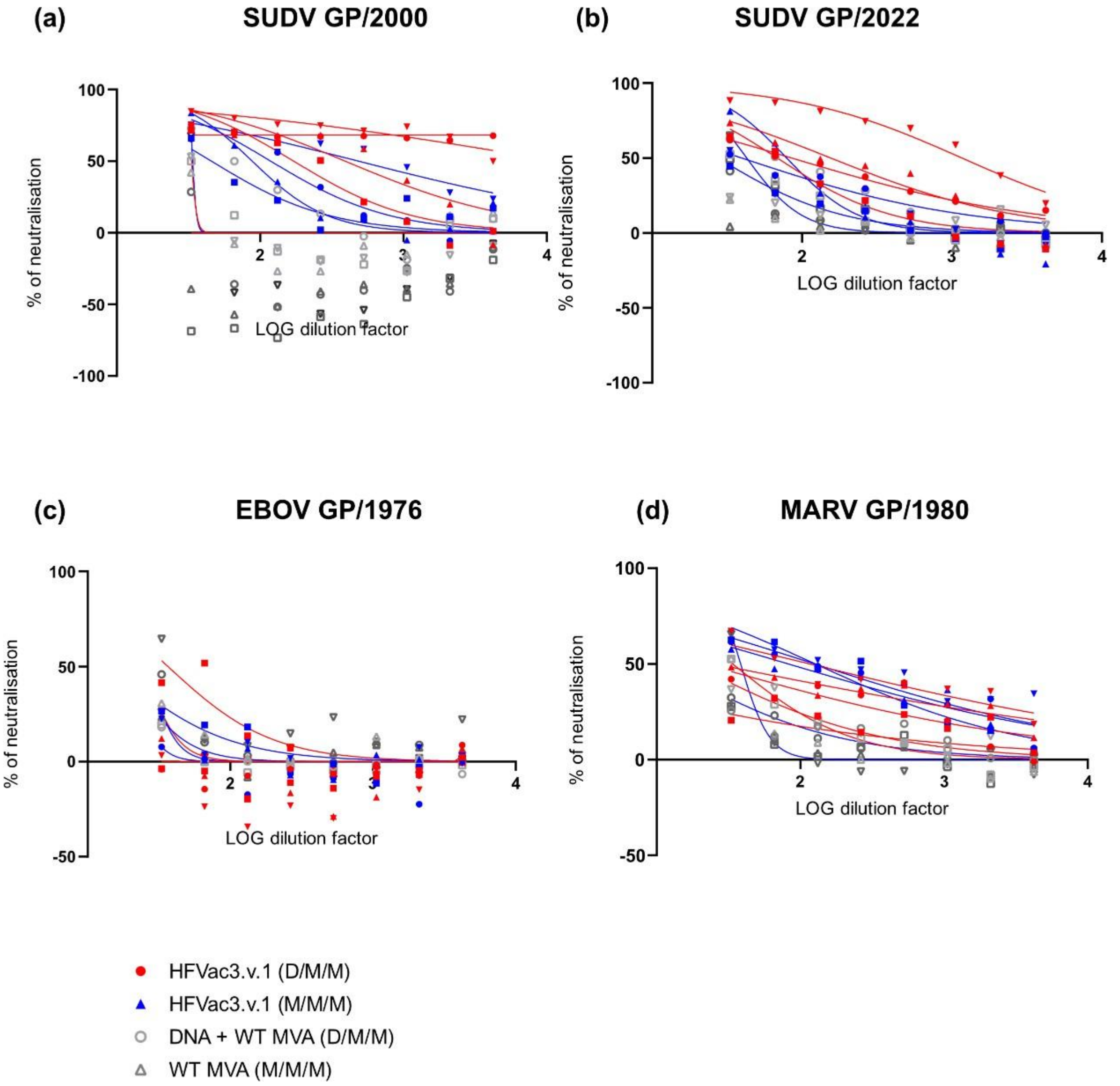

Supplementary Figure S9. Neutralizing antibody response in Hartley guinea pigs.

Supplementary to Fig.5 where neutralization is shown as IC50 values. Neutralizing antibodies in Hartley guinea pigs (n=4) measured two weeks after the third immunization using lentiviruses pseudotyped with glycoproteins of the indicated filoviruses **(a)** SUDV GP/2000 , **(b)** SUDV GP/2022 , **(c)** EBOV GP/1976, **(d)** MARV GP/1980. Each neutralization curve represents one animal at a certain serum dilution.

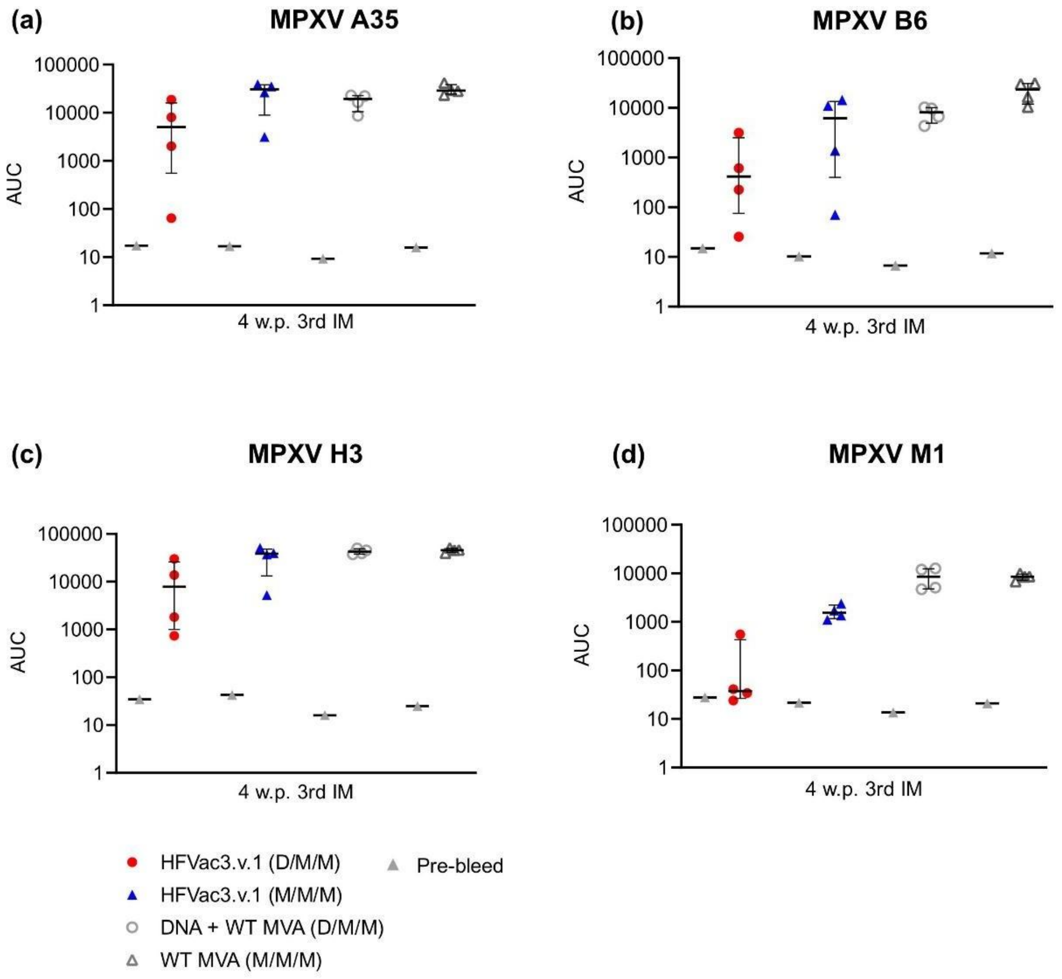

Supplementary Figure S10. Binding antibodies raised in Hartley guinea pigs against different MPXV proteins.

Hartley guinea pigs were vaccinated with the trivalent HFVac3.1 vaccines either in a heterologous (D/M/M; group G1) or homologous (M/M/M; group G2) immunization regimen. As controls empty DNA or WT MVA (group G3 and G4) were used, respectively. (a-d) Binding antibodies were analysed from the terminal bleeds using Luminex for the indicated surface MPXV proteins of mature and extracellular virions. Median responses and interquartile ranges are indicated, and each symbol represents an individual animal. Pre-bleeds of each group are depicted as grey triangles. AUC: Area under the curve. Statistical results were calculated using Mann-Whitney U-tests with * - $p < 0.05$; **- $p < 0.01$. w.p. x IM – weeks post x immunization.

Supplementary Table 1: Sensitivity, specificity, and accuracy of the Luminex assay for the specified antigens when analysing serum samples from mice.

Target	No. of negative samples testing positive (n=36) at the highest likelihood ratio	% specificity	95 % CI	No. of positive samples testing negative (n=36) at the highest likelihood ratio (n=36)	% sensitivity	95 % CI	% accuracy	95 % CI
EBOV GP	3	91.67	78.17-97.13	4	88.89	74.69-95.59	94.60	89.66-99.54
SUDV GP	0	100.00	90.36-100.00	6	86.11	71.34-93.92	94.87	88.87-100.00
MARV GP	1	97.22	85.83-99.86	5	86.11	71.34-93.92	97.49	94.46-100.00
RAVV GP	1	97.22	85.83-99.86	3	91.67	78.17-97.13	96.80	92.63-100.00
LASV NP	0	100.00	91.24-100.00	0	100.00	91.24-100.00	100.00	100.00-100.00

Supplementary Table 2: Sensitivity, specificity, and accuracy of the Luminex assay for the specified antigens when analysing serum samples from guinea pigs.

Target	No. of negative samples testing positive at the highest likelihood ratio (n=40)	% specificity	95% CI	No. of positive samples testing negative at the highest likelihood ratio (n=40)	% sensitivity	95% CI	% accuracy	95 % CI
EBOV GP	0	100.00	91.24-100.00	0	100.00	91.03-100.00	100.00	100.00-100.00
SUDV GP	0	100.00	91.24-100.00	1	97.44	86.82-99.87	99.68	98.96-100.00
MARV GP	0	100.00	91.24-100.00	0	100.00	91.03-100.00	100.00	100.00-100.00
RAVV GP	0	100.00	91.24-100.00	0	100.00	91.03-100.00	100.00	100.00-100.00
LASV NP	0	100.00	91.24-100.00	0	100.00	91.03-100.00	100.00	100.00-100.00
Mpox A35	1	97.06	85.08-99.85	0	100.00	90.82-100.00	100.00	100.00-100.00
Mpox M1R	1	97.06	85.08-99.85	0	100.00	90.82-100.00	98.84	96.51-100.00
Mpox B6R	1	97.06	85.08-99.85	0	100.00	90.82-100.00	100.00	100.00-100.00
Mpox H3L	1	97.06	85.08-99.85	0	100.00	90.82-100.00	100.00	100.00-100.00
